# Characterising inequalities in accessing primary care psychological therapies services for people living with dementia: the example of NHS talking therapies in England

**DOI:** 10.1017/S2045796025100309

**Published:** 2025-12-12

**Authors:** Celine El Baou, Rob Saunders, Joshua Eusty Jonathan Buckman, Marcus Richards, Claudia Cooper, Natalie Marchant, Roopal Desai, Georgia Bell, Caroline Fearn, Stephen Pilling, Nikki Zimmermann, Valerie Mansfield, Sebastian Crutch, Emilie V. Brotherhood, Amber John, Joshua Stott

**Affiliations:** 1 Research Department of Clinical, Educational and Health Psychology, UCL, London, UK; 2 Centre for Outcomes Research and Effectiveness, Research Department of Clinical, Educational and Health Psychology, UCL, London, UK; 3 iCope, Camden & Islington NHS Foundation Trust, London, UK; 4 MRC Unit for Lifelong Health and Ageing at UCL, UCL, London, UK; 5 Centre for Psychiatry and Mental Health, Wolfson Institute of Population Health, Queen Mary University, London, UK; 6 Division of Psychiatry, UCL, London, UK; 7 Camden & Islington NHS Foundation Trust, London, UK; 8 Dementia Research Centre, UCL Queen Square Institute of Neurology, London, UK

**Keywords:** dementia, mental health, primary care, psychotherapy, psychiatric services

## Abstract

**Aims:**

In England, psychological therapies provided in primary care are recommended as first-line treatment for people living with mild-to-moderate dementia experiencing depression or anxiety. It is known that people living with dementia experience more barriers to accessing therapy than people without dementia, but such inequalities in terms of rates of access to primary care services are yet to be characterised.

**Methods:**

In this retrospective, observational study of linked electronic healthcare records, the national database of the National Health Service (NHS) Talking Therapies for anxiety and depression programme was used to compare pathways to accessing therapy between 6623 people living with dementia and 4 825 489 without dementia between 2012 and 2019. Outcomes included access to an assessment, to therapy and reasons for discharge. Primary analyses used a propensity-score matched cohort to compare outcomes. Exact matching was used for the NHS service entity.

**Results:**

The prevalence of dementia in the study cohort was lower than the prevalence of dementia in a representative population, based on an estimation of prevalence in people with mild-to-moderate age over 35 (0.23% in our study vs 3.82% in previous research). Compared to people without dementia, people living with dementia were less likely to access an assessment (odds ratio [OR] = 0.60; 95% confidence interval [CI]: 0.55–0.65), to subsequently receive therapy (OR = 0.67; 95% CI: 0.61–0.73) and more likely to be discharged because services were deemed not suitable before having an assessment (relative rate ratio [RRR] = 4.90; 95% CI: 4.20–5.72) and starting therapy (RRR = 2.74; 95% CI: 2.24–3.35). Female gender, social deprivation, Asian ethnicity and less common dementia subtypes (such as frontotemporal dementia) were also associated with poorer access rates and a higher likelihood of services being deemed not suitable. Involvement of care partners in the referral process was associated with better access rates.

**Conclusions:**

Pathways to accessing primary care psychological therapy services must be made more accessible for people living with dementia. Better access could be achieved by increasing referrer awareness and training for staff within services to promote access for people living with dementia (especially for groups under-represented in services), better understanding how to involve care partners in the process, as well as when specialist support might be more suited in secondary care. More granularity in the medical coding of rarer dementia diagnoses in electronic health records would also allow for better statistically powered research for these groups.

## Introduction

Dementia is a leading cause of death worldwide (Gauthier *et al.*, 2021), contributing significantly to the Global Burden of Disease, with an estimated 1.62 million deaths attributed to dementia globally in 2019 (Gbd 2019 Mental Disorders Collaborators, 2022). It is estimated that more than 55 million people globally (World Health Organisation, 2023) and 944 000 adults in the UK (Prince *et al.*, 2013) are living with dementia, making dementia prevention, treatment and care a World Health Organization public health priority (World Health Organization, 2021).

Dementia is an umbrella term (Gauthier *et al.*, 2021) for a syndrome caused by neurodegeneration associated with a decline in neurological and cognitive functioning. Amnestic Alzheimer’s disease, characterised initially by episodic memory decline, is its most common subtype. Less common subtypes, such as frontotemporal dementia (FTD) (Marshall *et al.*, 2018; Piguet *et al.*, 2011), are typically characterised by initial difficulties with speech, language or personality changes. These less common subtypes of dementia are also associated with a younger age of onset (before the age of 65), which gives rise to specific needs in terms of care and social support (Greenwood and Smith, 2016; Millenaar *et al.*, 2016).

People living with dementia have a lower chance of receiving physical or mental health care than people without dementia (Cooper *et al.*, 2016). This is compounded by further characteristics associated with poorer access to healthcare, including gender, ethnicity, social deprivation and geographical location (e.g. urban vs rural setting), which affect access to diagnostic assessments for dementia and access to healthcare post-diagnosis (Giebel *et al.*, 2021; Watson *et al.*, 2021, 2023).

People living with mild-to-moderate dementia are more likely to experience anxiety and depression than people without dementia (estimated point prevalence of depression is ∼40.0% among people with mild-to-moderate dementia over the age of 35 [Leung *et al.*, 2021] and 12.9% for the general adult population [Lim *et al.*, 2018]). Evidence-based psychological therapies, such as cognitive–behavioural therapies, are recommended and potentially beneficial first-line interventions for common mental health problems for people living with mild-to-moderate dementia (Bell *et al.*, 2022). However, this population faces considerable barriers in accessing psychological therapies despite a general preference for psychological over pharmacological treatment (Mchugh *et al.*, 2013) and evident motivation to engage in therapy from both people living with dementia and their clinicians (Baker *et al.*, 2022). Access to therapy is more challenging for people living with dementia because of stigma towards older adults, and because some referrers do not recognise that people with dementia can benefit from talking therapies (Baker *et al.*, 2022). However, socio-demographic factors associated with such inequalities have not been characterised, and prior studies have not investigated whether those with different dementia subtypes have differential pathways into and out of psychological therapies for common mental disorders at the national level.

In England, in the National Health Service (NHS), the majority of evidence-based psychological therapies are provided through primary care psychological therapy services, NHS Talking Therapies for anxiety and depression (NHS TTad) (NHS Digital, 2021b), and with such services recommended as first-line treatment for people living with mild to moderate dementia with co-occurring depression or anxiety (NICE, 2018). Making such services accessible for people living with mild-to-moderate dementia is part of the NHS long-term plan (Department of Health, 2019). This study uses electronic healthcare records with national coverage in England to investigate whether there are potential inequalities in treatment pathways of people living with dementia to the NHS TTad programme, and whether any access limitations are exacerbated by existing inequalities, with the aim to:

- Identify whether pathways to accessing psychological therapy differ between people living with dementia and people living without dementia.
- Examine which demographic factors (age, gender, ethnicity, index of deprivation) may be associated with accessing psychological therapy for people living with dementia.

## Methods

Analyses were reported following REporting of studies Conducted using Observational Routinely-collected Data principles (Benchimol *et al.*, 2015) (RECORD) (see Supplement for checklist). Analyses were conducted using Stata 17.

### Databases

The MODIFY dataset was used (Bell *et al.*, 2022; John *et al.*, 2023). It includes linked electronic healthcare records from four databases of national coverage: the NHS TTad database (NHS Digital, 2021b), the Hospital Episode Statistics database (NHS Digital, 2019), the HES-ONS mortality database (NHS Digital, 2023) and the Mental Health Services Dataset (NHS Digital, 2021a). Individual records were linked at the person-level using an anonymous linkage key (see Supplement A).

### Procedures

Psychological therapies are delivered in NHS Talking Therapies for anxiety and depression by NHS-accredited practitioners (National Collaborating Centre for Mental Health, 2025). Some practitioners may have received dementia-specific awareness as part of their training, but it is not known to what extent they may have been trained to work specifically with people living with dementia, beyond information available in clinical guidelines (Baker *et al.*, 2022; NICE, 2018) (Supplement B).

Access to a referral is open to anyone experiencing difficulties with their mental health. Information about the referral and assessment process is available in Supplement B.

People referred to NHS TTad services may withdraw or opt out at any point, with no obligation to provide a reason.

### Study population

**Figure 1.**
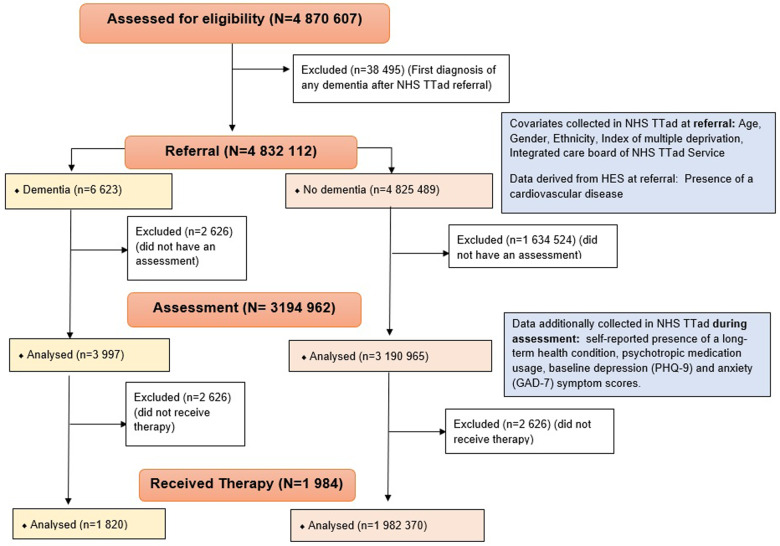
Study flow chart.

The study population included 4 832 112 NHS TTad attendees aged over 18 who were referred to any NHS TTad service between 2012 and 2019 and met any of the dementia ascertainment definitions, including 6623 people living with dementia before referral. A study flow chart is available in Figure 1. For people who were referred to services several times, the first referral was retained.

### Outcomes

Treatment pathways were examined by calculating the proportion of people who accessed NHS TTad services at two time points in their treatment journey: assessment and start of therapy.

*Assessment access rates* were calculated as the proportion of people referred to NHS TTad services who received an assessment as described above.

*Therapy access rates* were calculated as the proportion of people who entered a course of therapy among those who received an assessment.

**Figure 2.**
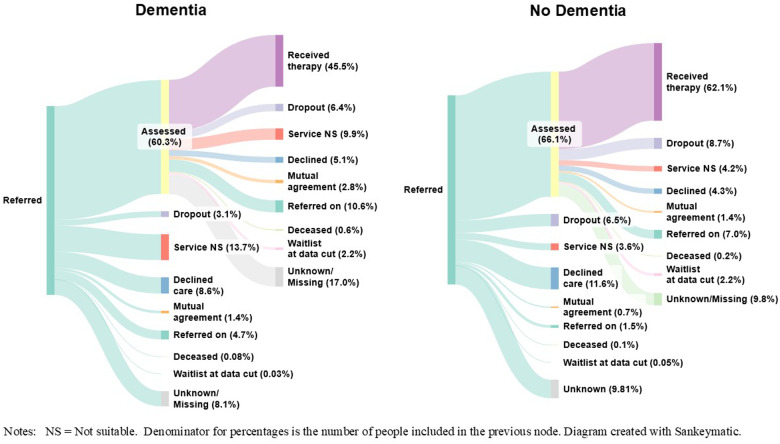
Treatment pathways for people living with, or without dementia.

*Reasons for discharge* were evaluated between referral and assessment (i.e., reasons for not receiving an assessment), and between assessment and start of therapy (i.e., reasons for not receiving therapy) (see Figure 2 and Supplement C for more details).

### Dementia ascertainment

Dementia code ascertainment was carried out based on the procedure described in our previous study using the same data source (El Baou *et al.*, 2024), using code lists based on the International Classification of Diseases 10th Revision (ICD-10) (see supplement D).

### Covariates

Covariates were included in the analyses on the basis of being potentially associated with the risk of dementia and with the study outcomes (access to assessment and to therapy).

For analyses of access to assessments, variables available at referral were included as covariates: age, gender, ethnicity, and index of multiple deprivation (Buckman *et al.*, 2021, 2023; Cooper *et al.*, 2016; Saunders *et al.*, 2021). Presence of cardiovascular (Brain *et al.*, 2023) or cerebrovascular disease was also adjusted for in the analyses, and identified using the following ICD-10 codes in the HES database: I20–25, I26–I28, I30–I52, I60–I69 and I70–79 (Huusko *et al.*, 2020).

For analyses of access to therapy, variables additionally collected during the assessment were also included as covariates: self-reported psychotropic medication use, baseline depression (using the PHQ-9 [Kroenke and Spitzer Robert, 2002]) and anxiety severity (using the GAD-7 [Spitzer *et al.*, 2006]), self-reported presence of a long-term health condition (Panchal *et al.*, 2020) (see Figure 1 for more details). The PHQ-9 and the GAD-7 have been validated in populations that likely comprise many people with dementia (e.g., nursing homes or people with neurological disorders), and are routinely used in NHS TTAd services and in previous dementia research (Bélanger *et al.*, 2019; Sun *et al.*, 2022).

To evaluate potential service level and geographical disparities, a measure of local NHS TTad entity (NHS integrated care board or ICB) was also used in the models (see Supplement E).

### Statistical analyses

1. Summary statistics of demographic characteristics for people living with or without dementia. Dementia prevalence in the sample was also compared to available estimates of depression and anxiety prevalence in dementia and general populations (Clark, 2018; Collins *et al.*, 2023; Wittenberg *et al.*, 2019) to obtain an idea of the representation of people living with dementia among people referred to psychology primary care services.
2. Examination of whether having a dementia diagnosis (vs no dementia diagnosis) was associated with a lower likelihood of being seen for an assessment or subsequently entering therapy. Logistic regression models were fitted, including dementia diagnosis (yes/no) as a covariate (Model 1), additionally adjusted for covariates previously described (Model 2). To consider that demographic characteristics may differ considerably between people with or without a dementia diagnosis, propensity score matching was used to identify a group of people without a dementia diagnosis matched on demographic characteristics with the cohort of people with a dementia diagnosis (Model 3). Such a method is also recommended because the size of the comparison group is disproportionately higher than the size of the dementia group (Williamson and Forbes, 2014).

For this last model, a propensity score (estimating the probability of having a dementia diagnosis vs no dementia diagnosis), was estimated using logistic regression, including all factors possibly associated with the outcomes as covariates as defined above. To account for geographical or service-level variations in service provision within the NHS, exact matching was used for the NHS TTAd service entity (ICB) to which people were referred to (see Supplement F). Model 3, accounting for the most bias correction, was considered the primary model.

1. Multinomial logistic regressions were run to evaluate whether having a dementia diagnosis (vs no dementia diagnosis) was associated with one of 7 possible different reasons for discharge at each stage (i.e., before receiving an assessment, or before entering therapy), using the same covariates and specifications as in Models 1–3 outlined above. Due to small sample sizes in the dementia group, people whose reason for discharge was ‘deceased’ or ‘ongoing at data cut-off’ were excluded from these analyses.
2. Multilevel logistic regressions were used to determine factors associated with being seen for an assessment or subsequently receiving therapy amongst the cohort of people living with dementia. All available covariates were included as fixed effects. NHS TTad service was included as a random effect (Model 4).

Finally, to better understand disparities by subtype within the dementia population, supplementary analyses were also conducted to examine whether subtypes of dementia are associated with different treatment pathways, and whether this differs by age group, with the caveat that such analyses may have less statistical power due to the lower prevalence of non-memory-led dementia presentations, such as FTD. These analyses are detailed in Supplement G.

### Missing data

A ‘missing’ data category was included in analyses for categorical variables as in previous research (Bell *et al.*, 2022; El Baou *et al.*, 2023; Saunders *et al.*, 2021), so as to avoid imputing data for protected demographic characteristics, such as ethnicity.

### Patient public involvement

Two patient public involvement (PPI) representatives (N.M. and V.M.) were involved in this project and attended regular meetings during the conduct of the study. They confirmed that the aims of the project fit with PPI priorities and provided suggestions of potential covariates to be used in analyses (mode of referral and geographical considerations such as NHS TTad service entity and region). They will be involved in co-designing materials to disseminate the findings of this research to the wider public.

## Results

Descriptive statistics for demographic characteristics and outcomes are presented in Table 1. Of those referred to NHS TTad services, we identified 6623 people living with all-cause dementia, and 4 825 489 people without a diagnosis of dementia in their healthcare record. A higher proportion of people living with all-cause dementia were reported to be male, older and to have a cardiovascular disease prior to referral than people living without dementia. To evaluate the representativeness of people living with dementia being referred to NHS TTad, we calculated the prevalence of dementia in the full study cohort and in a subset of people over 35 years old, to compare prevalence in our cohort to prevalence in a previous study conducted in this age group (Wittenberg *et al.*, 2019). We found that 0.14% of the study cohort and 0.23% of those over 35 years old had a diagnosis of dementia. For comparison, and assuming prevalence of depression in mild-to-moderate dementia (38.0%) and general population (12.9%) from previous studies (Collins *et al.*, 2023; Wittenberg *et al.*, 2019), we estimated the expected prevalence of mild-to-moderate dementia in a representative population of people experiencing depression over 35 years old to be 3.82% (see Supplement H for calculation).

**Table 1.** Demographic characteristics and outcomes

	All-cause dementia	No dementia
Demographic and baseline measures	*N* = 6623	*N* = 4 825 489
Age at referral, mean (SD) range	67 (16.7)	39.6 (15.1)
Age category, *n* (%)		
18–34	351 (5.3%)	2 110 165 (43.7%)
35–64	2090 (31.6%)	2 386 674 (49.5%)
65+	4182 (63.1%)	328 650 (6.8%)
Ethnicity		
White	4172 (63.0%)	3 309 999 (68.6%)
Mixed	59 (0.89%)	85 990 (1.8%)
Asian	285 (4.3%)	199 838 (4.1%)
Black	179 (2.7%)	116 809 (2.4%)
Other	60 (0.91%)	51 558 (1.1%)
Missing	1868 (28.2%)	1 061 295 (22.0%)
Gender		
Male	2746 (41.5%)	1 670 355 (34.6%)
Female	3778 (57.0%)	3 107 377 (64.4%)
Missing	99 (1.5%)	47 757 (0.99%)
Index of multiple deprivation (IMD) Quintile, *n* (%)		
1 (most deprived)	1652 (24.9%)	1 140 104 (23.6%)
2	1384 (20.9%)	1 023 366 (21.2%)
3	1297 (19.6%)	898 021 (18.6%)
4	1080 (16.3%)	810 540 (16.8%)
5 (least deprived)	956 (14.4%)	716 801 (14.9%)
Missing	254 (3.8%)	236 657 (4.9%)
Presence of cardiovascular disease before referral, *n* (%)	3049 (46.0%)	209 329 (4.3%)
Source of referral, *n* (%)		
General practitioner/primary care services	3020 (45.6%)	2 120 461 (43.9%)
Self-referral	2482 (37.5%)	2 338 084 (48.5%)
Referred by carer	88 (1.33%)	1380 (0.03%)
Other	1033 (15.6%)	365 564 (7.6%)
Outcome: % assessed		
N (%) assessed	3997 (60.3%)	3 190 965 (66.1%)
N (%) not assessed Reason	2626 (39.7%)	1 634 521 (33.9%)
Dropout (unscheduled discontinuation)	202 (3.1%)	312 239 (6.5%)
Service not suitable	910 (13.7%)	175 364 (3.6%)
Person declined care	568 (8.6%)	561 307 (11.6%)
Mutual agreement and signposting	95 (1.43%)	34 244 (0.71%)
Referred on	311 (4.7%)	72 840 (1.5%)
Deceased	5 (0.08%)	4 370 (0.09%)
Waiting for assessment at data-cut	2 (0.03%)	2614 (0.05%)
Missing	533 (8.1%)	471 546 (9.8%)
**Outcome: % received therapy**	***N* = 6623**	***N* = 4 825 489**
N assessed (denominator)	3 997	3 190 965
N (%) received therapy Reason	1820 (45.5%)	1 982 370 (62.1%)
Dropout (unscheduled discontinuation)	254 (6.4%)	278 622 (8.7%)
Service not suitable	396 (9.9%)	135 456 (4.2%)
Person declined care	204 (5.1%)	138 856 (4.3%)
Mutual agreement and signposting	111 (2.8%)	45 651 (1.4%)
Referred on	424 (10.6%)	222 102 (7.0%)
Deceased	25 (0.63%)	5 676 (0.18%)
Waiting for start of therapy at data-cut	88 (2.2%)	68 981 (2.2%)
Missing	675 (17.0%)	313 251 (9.8%)

SD = standard deviation.

### Comparisons of treatment pathways between people living with all-cause dementia and people living without dementia

Treatment pathways from referral through to receiving treatment are presented in Figure 2.

Of people living with dementia who were referred to NHS TTad services, 60.3% received an assessment. Of those, 45.5% then received therapy, meaning that overall, 27.5% of people living with dementia who were referred to services received a course of therapy.

Out of people without a diagnosis of dementia who were referred to NHS TTad services, 66.1% received an assessment. Of those, 62.1% went on to receive therapy, meaning that overall, 41.1% of people without a diagnosis of dementia received a course of therapy.

#### Access to assessment

In the matched analyses using simple logistic regression (Table 2), the odds of receiving an assessment were 40% lower for people living with dementia than for the comparison group (Model 3: odds ratio [OR] = 0.60; 95% CI: 0.55–0.65; *P* < 0.000). In the matched analyses using multinomial logistic regression, differences also appeared in reasons for discharge, as people living with dementia were 3.3 more likely to be referred on to further services vs being assessed than people without (Model 3 relative rate ratio [RRR] = 3.30; 95% CI: 2.64–4.12; *P* < 0.0001), 4.9 times more likely for the service to be deemed unsuitable (Model 3 RRR = 4.90; 95% CI: 4.20–5.72; *P* < 0.0001) and 1.69 more likely to be discharged following mutual agreement and signposting (Model 3 RRR = 1.69; 95% CI: 4.24–1.31; *P* = 0.0009) than people without dementia.

**Table 2.** Comparisons of outcomes for people living with all-cause dementia vs no dementia

All cause dementia vs no dementia	Model 1: Unadjusted			Model 2: Adjusted			Model 3: Matched cohort	
Outcome: Receiving an assessment – Referral cohort *N* = 4 832 112								
N included in analyses	N with dementia = 6623 N with no dementia = 4 825 489					N with dementia = 6623 N with no dementia = 6623		
Receiving an assessment – Simple logistic regression – OR (95% CI) *P*-value								
Assessed (ref = No)	0.78 (0.74; 0.82)	<0.0001	0.57 (0.54; 0.60)		<0.0001	0.60 (0.55; 0.65)		<0.0001
Reason for not receiving an assessment – Multinomial logistic regression – RRR (95% CI) *P*-value								
Completed (ref)	–		–			**–**		
Dropout (unscheduled discontinuation)	0.52 (0.45; 0.59)	<0.0001	0.92 (0.80; 1.06)		0.2597	0.93 (0.77; 1.13)		0.4878
Service not suitable	4.14 (3.85; 4.45)	<0.0001	4.98 (4.61; 5.39)		<0.0001	4.90 (4.20; 5.72)		<0.0001
Person declined care	0.81 (0.74; 0.88)	<0.0001	1.05 (0.96; 1.16)		0.2666	0.99 (0.87; 1.12)		0.8716
Mutual agreement and signposting	2.21 (1.81; 2.72)	<0.0001	1.61 (1.31; 1.99)		<0.0001	1.69 (1.24; 2.31)		0.0009
Referred on	3.41 (3.04; 3.83)	<0.0001	3.84 (3.41; 4.33)		<0.0001	3.30 (2.64; 4.12)		<0.0001
Missing	0.90 (0.82; 0.99)	0.0260	1.22 (1.11; 1.34)		<0.0001	1.24 (1.09; 1.42)		0.0010
Outcome: Receiving therapy – Assessment cohort *N* = 3 194 962								
Receiving therapy – Simple logistic regression – OR (95% CI) *P*-value								
N included in analyses	M with dementia = 3 997 N without dementia = 3 190 965					N with dementia = 3997 N without dementia = 3997		
Received therapy (ref = No)	0.51 (0.49; 0.54)	<0.0001	0.66 (0.62; 0.71)		<0.0001	0.67 (0.61; 0.73)		<0.0001
Reason for not receiving treatment – Multinomial logistic regression – RRR (95% CI) *P*-value								
Completed (ref)	–		–					
Dropout (unscheduled discontinuation)	0.99 (0.87; 1.13)	0.9160	1.14 (1.00; 1.32)		0.0499	1.12 (0.93; 1.36)		0.2432
Service not suitable	3.18 (2.86; 3.55)	<0.0001	2.42 (2.15; 2.72)		<0.0001	2.74 (2.24; 3.35)		<0.0001
Person declined care	1.60 (1.38; 1.85)	<0.0001	1.24 (1.06; 1.44)		0.0064	1.24 (1.00; 1.54)		0.0532
Mutual agreement and signposting	2.65 (2.19; 3.21)	<0.0001	1.17 (0.95; 1.45)		0.1394	1.14 (0.86; 1.52)		0.3673
Referred on	2.07 (1.87; 2.31)	<0.0001	1.77 (1.58; 1.98)		<0.0001	1.86 (1.56; 2.21)		<0.0001
Missing	2.35 (2.14; 2.56)	<0.0001	1.31 (1.18; 1.46)		<0.0001	1.34 (1.15; 1.56)		0.0002

Adjusted models include age group, gender, ethnicity, IMD quintile, presence of a cardiovascular disease before referral and NHS integrated care board as fixed effect for both cohorts, and are additionally adjusted for variables collected during the assessment in the assessment cohort: taking psychotropics at assessment, presence of a self-reported health conditions and depression and anxiety severity symptom scores. People living with dementia were matched 1:1 without replacement to an individual without a diagnosis of dementia. A match was achieved for each individual. Robust standard errors were used in the matched analyses to account for the fact that the propensity score was estimated. People (*N* = 6991) whose reasons for discharge were ‘deceased’ and ‘ongoing at data cut-off’ were excluded from the analyses due to small sample sizes in the dementia group.

OR = odds ratio; RRR = relative rate ratio.

#### Access to therapy

Similarly, compared to people without a diagnosis of dementia, the odds of receiving therapy were lower for people living with dementia (Model 3: OR = 0.67 [0.61–0.73]; *P* < 0.0001). People living with dementia were also more likely not to receive treatment because they were referred on to another service (Model 3 RRR = 1.86 [1.56–2.21]; *P* < 0.0001) and for the service to be deemed unsuitable (Model 3 RRR = 2.74 [2.24–3.35]; *P* < 0.0001).

### Factors associated with access to psychological therapy for people living with dementia

#### Demographic disparities

For people living with dementia, being of Asian ethnicity (vs White), living in the most (vs least) deprived areas, and being referred by general primary care services (vs other referral pathways) were associated with lower odds of both being seen for an assessment and subsequently receiving therapy (Table 3). Being female (vs male) was associated with lower odds of being seen for an assessment only, and having cardiovascular disease or mild anxiety symptoms (vs moderate to severe) was associated with lower odds of receiving therapy.

**Table 3.** Factors associated with access to psychological therapy for people living with dementia

	% receiving an assessment			% entering therapy		
Factor	%	Multilevel model OR (95% CI)	*P*-value	*N* (%)	Multilevel model OR (95% CI)	*P*-value
Age, ref ≤65	60.6%	ref		49.4%	ref	
≥65	60.2%	1.02 (0.90–1.15)	0.7174	43.3%	0.92 (0.79–1.07)	0.2771
Gender, ref = Male	62.7%	ref		44.4%	ref	
Female	58.8%	0.84 (0.75–0.94)	0.0026	46.9%	1.08 (0.94–1.24)	0.2542
Missing	53.5%	1.46 (0.93–2.30)	0.0998	40.8%	1.06 (0.49–2.29)	0.8736
Ethnicity, ref = White	69.6%	ref		48.6%	ref	
Mixed	71.2%	1.05 (0.59–1.95)	0.8000	52.4%	1.14 (0.60–2.17)	0.6871
Asian	69.5%	0.71 (0.53–0.94)	0.0186	37.9%	0.61 (0.44–0.86)	0.0038
Black	77.1%	0.99 (0.68–1.45)	0.9758	47.8%	1.00 (0.68–1.48)	0.9783
Other	73.3%	1.00 (0.55–1.82)	0.9903	38.6%	0.67 (0.35–1.28)	0.2248
Missing	35.9%	0.23 (0.20–0.26)	<0.0001	34.2%	0.75 (0.61–0.93)	0.0082
IMD quintile, ref = 1st (most deprived)	59.9%	ref		44.1%	ref	
2nd	61.6%	1.11 (0.94–1.32)	0.1973	44.0%	1.12 (0.91–1.37)	0.2800
3rd	59.7%	1.12 (0.95–1.33)	0.1837	47.3%	1.32 (1.07–1.63)	0.0103
4th	61.1%	1.23 (1.03–1.48)	0.0249	45.9%	1.28 (1.02–1.60)	0.0318
5th (least deprived)	58.5%	1.19 (0.98–1.45)	0.0758	49.6%	1.46 (1.15–1.86)	0.0023
Missing	64.2%	1.30 (1.96–1.78)	0.0930	38.7%	1.18 (0.80–1.72)	0.4054
Cardiovascular disease at referral, ref = No	59.7%	ref		48.2%	ref	
Yes	61.1%	1.07 (0.95–1.20)	0.2549	42.5%	0.86 (0.75–0.99)	0.0400
Source of referral						
Primary care	49.7%	ref		47.5%	ref	
Carer	93.2%	5.38 (2.20–13.16)	0.0002	89.0%	9.70 (4.42–21.28)	<.0001
Self-referral	72.0%	1.83 (1.60–2.08)	<0.0001	41.0%	1.26 (1.02–1.54)	0.0292
Other (e.g., secondary care)	60.9%	1.45 (1.24–1.70)	<0.0001	48.2%	0.75 (0.64–0.87)	0.0002
Taking psychotropic medications at referral, ref = No	Not applicable, covariates collected during assessment			47.0%	ref	0.5816
Yes				49.3%	0.96 (0.82–1.12)	0.0868
Missing				36.6%	0.83 (0.68–1.03)	
Self-reported presence of a long-term health condition, ref = No				48.5%	ref	
Yes				47.8%	0.91 (0.77–1.08)	0.2974
Missing				39.8%	0.80 (0.65–0.99)	0.0377
Depression score severity						
Depression, ref = Mild (PHQ − 9 < 15)				45.6%	ref	
Moderate to severe (PHQ − 9 ≥ 15)				51.7%	0.97 (0.82–1.14)	0.6925
Missing				14.5%	2.25 (0.34–15.00)	0.4036
Anxiety score severity						
Anxiety, ref = Mild (GAD − 7 < 10)				40.8%	ref	
Moderate to severe (PGAD − 7 ≥ 10)				5.9%	1.89 (1.59–2.24)	<0.0001
Missing				14.0%	0.15 (0.02–1.03)	0.0539

#### Local disparities

In multilevel models, we estimated that NHS TTad local entity clustering represented 7.3% (intra-class correlation coefficient [ICC] = 0.073; 95% CI: 0.045–0.116) and 5.8% (ICC = 0.058; 95% CI: 0.033–0.099) of the variability in access to assessment and therapy, respectively, as measured by the ICC, suggesting that local disparities may influence access to assessment and therapy, independently of other factors (see Supplement I).

#### Supplementary analyses: Dementia subtype disparities

Supplementary analyses across dementia subtypes revealed that the likelihood of receiving an assessment was the lowest for people living with FTD, compared to all other subtypes. Differences between subtypes in terms of the likelihood of receiving were less salient, although less statistical power was available in these analyses (see Supplement G).

In addition, people under the age of 65 with combined dementia presentations, PD dementia, atypical AD, and FTD had the lowest rates of access to both assessment and treatment and were the most likely to be discharged because the NHS TTad service was deemed ‘not suitable’ (see Supplement G for more details).

## Discussion

This study used a cohort of national coverage to characterise inequalities in access to primary care psychological therapy services for people living with dementia in England. Indeed, we found that people with dementia who were referred or self-referred to talking therapies were less likely to be assessed for treatment, and to subsequently enter therapy than people living without dementia. This was because they were more frequently referred to other services, or because the services were deemed unsuitable to support them. Given that psychological therapy services are the first-line recommended treatment in England for depression and anxiety, it suggests that people living with dementia seeking support for their mental health may face more barriers to accessing such support, or additional delays in receiving care if they are subsequently referred to services in secondary care, where they would have to start over the assessment process. This study is in line with previous research highlighting challenging pathways to healthcare for people living with dementia (Cooper *et al.*, 2016), and is, to our knowledge, the first to quantify these inequalities in accessing talking therapies, and to do so in national data.

This study also highlighted specific inequalities in access to care amongst people living with dementia. Younger people with complex presentations or non-memory-led presentations were the least likely to be seen by services, which is consistent with previous qualitative research highlighting the unmet needs of people living with younger-onset dementia in their care-seeking journey (Millenaar *et al.*, 2016). Consistent with findings of previous dementia research on access to general primary care services, female gender was associated with a lower likelihood of accessing services (Cooper *et al.*, 2015). People living with dementia from more socially deprived areas were also less likely to access services, which constitutes a new finding since social deprivation was not previously associated with lower access to general primary care services (Cooper *et al.*, 2015). People of Asian ethnicities were also less likely to access services than people from White ethnicities, which may mirror findings from previous research highlighting the specific barriers to healthcare experienced by UK South Asian people living with dementia (Blakemore *et al.*, 2018), who make up a large proportion of this group in our cohort. This may reflect a known under-utilisation of mental health services by UK South Asian communities (Prajapati and Liebling, 2022), language barriers, a lack of culturally adapted interventions, and cultural perceptions of dementia in these communities (Giebel *et al.*, 2019). Further research is warranted to understand how age, gender, ethnicity and social deprivation may affect the experiences of accessing psychological therapy services.

In addition, people living with dementia were more likely to access an assessment if they self-referred or were referred to services by their care partners, as opposed to general primary care services, which underlines how important self-referral pathways are for people living with dementia. Together with the heterogeneity of access rates observed according to services and regions, this suggests a need to better understand how individual services may facilitate referral pathways at the local level for people living with dementia. Understanding the involvement of care partners or family members in the referral process is also an avenue for future research.

Finally, it is important to note that the prevalence of dementia in the study cohort was more than 10 times lower than expected in a representative population of people experiencing mental health difficulties, suggesting that the vast majority of people with mild-to-moderate dementia who experience depression or anxiety are not referred to, or do not self-refer to primary care psychological therapy services, the recommended first-line intervention in such cases. Even though it is not known whether or how many people living with dementia may access therapy through other routes (private counselling, secondary care services), this highlights a potentially important health inequality as this database has national coverage and could also suggest that the service pathway for people living with dementia who experience anxiety and depression needs to include a combination of primary care and specialist psychological therapy services.

Overall, using data of national coverage means that findings are generalisable to the population of people living with dementia seeking access to talking therapies in England, with the caveat that their dementia diagnosis must be identified in their electronic healthcare record.

### Limitations

Our study also has some important limitations. First, even though the Hospital Episode Statistics database showed good agreement to identify people living with common subtypes of dementia, such as Alzheimer’s disease and vascular dementia (Brown *et al.*, 2016), it is possible that some people living with, or without dementia, were misclassified in our analyses. Moreover, less is known about the reliability of this database for less common forms of dementia, such as FTD. This is especially relevant because ICD-10 codes do not allow for the identification of certain subtypes of dementia, such as Dementia with Lewy bodies, or of types of dementia associated with specific symptoms for language (such as primary progressive aphasia) and visual processing (such as posterior cortical atrophy) (Uysal, 2019). Such limitations may be partially addressed with the addition of specific codes in future versions of ICD classification (World Health Organization, 2025), but this lack of granularity in the coding system may translate into difficulties recording diagnoses in the care system and smaller sample sizes when considering analyses by dementia subtypes. For these reasons, and for issues associated with multiple testing, supplementary findings by dementia subtype should be considered exploratory, as the potential for spurious findings caused by multiplicity, low power, or measurement error cannot be ruled out.

Second, no information was available for dementia severity or for the symptoms experienced by people seeking psychological therapies, and no information was available about alternative services provided when services were deemed unsuitable. It is plausible that service providers in these general primary care services may lack awareness or confidence in working with people with dementia (Baker *et al.*, 2022). Moreover, this means our study cannot capture whether people with severe dementia were referred to services and could not receive therapy due to a lack of capacity to engage in the intervention. More research is required to understand how dementia symptoms may affect access to psychological therapy after referral, and how potential barriers caused by symptoms may be addressed by services.

Finally, these study findings are not generalisable to people living with dementia who are not referred or do not self-refer to talking therapies. More research is needed to understand whether this population is representative of the population of people living with dementia, and to examine whether specific inequalities appear before seeking referral to therapy.

## Conclusion

This study quantifies significant inequalities in accessing psychological therapies in primary care for people living with dementia across England. With plans to make services such as NHS TTAd in England more accessible to people living with dementia, it is of public health concern to understand how to address the pathways to assessment and on to therapy once services are accessed, and in which cases specialist support might be more suited in secondary care. Further, within the dementia population, the systemic inequalities highlighted in this study regarding gender, ethnicity, social deprivation, dementia subtype and presentation, as well as local-level disparities, suggest that such support needs to be tailored or potentially targeted at specific groups. Support could take many forms, but might include increasing referrer awareness that psychological services can be suitable for dementia and culturally sensitive training for staff within services to promote access for people living with dementia.

## Supporting information

10.1017/S2045796025100309.sm001 El Baou et al. supplementary material El Baou et al. supplementary material

## Data Availability

All data used for this study are available upon successful application to NHS Digital via the Data Access Request Service (DARS): https://digital.nhs.uk/services/data-access-request-service-dars.

## Supplementary material

The supplementary material for this article can be found at https://doi.org/10.1017/S2045796025100309.
