# Supplementary material for: Characterising inequalities in accessing primary care psychological therapies services for people living with dementia: the example of NHS talking therapies in England
Source: Epidemiol Psychiatr Sci. 2025 Dec 12;34:e60. doi: 10.1017/S2045796025100309 (PMC12721990; doi:10.1017/S2045796025100309)
Supplement: El Baou et al. supplementary material [file S2045796025100309sup001.docx]

**SUPPLEMENT**

[Supplement A MODIFY Study Dataset 2](#_Toc211865297)

[Supplement B NHS Talking Therapies for anxiety and depression Services 3](#_Toc211865298)

[Supplement C Reasons for discharge 5](#_Toc211865299)

[Supplement D Dementia code list 6](#_Toc211865300)

[Supplement E Covariates used in analyses 8](#_Toc211865301)

[Supplement F Matching Procedure 10](#_Toc211865302)

[Supplement G Supplementary analyses - Dementia Subtypes disparities 15](#_Toc211865303)

[Supplement H Expected prevalence of dementia in general depression population over 35 21](#_Toc211865304)

[Supplement I Local disparities 22](#_Toc211865305)

[Supplement J Authors contributions 23](#_Toc211865306)

[Supplement K The RECORD statement – checklist of items, applied to the present study 24](#_Toc211865307)

# MODIFY Study Dataset

| **NHS TTad/IAPT dataset  (National Collaborating Centre for Mental Health, 2021a)** | Routinely collected data for every patient seen in NHS TTAD services across all 211 clinical commissioning group areas in England between 2012 to 2019. Includes demographic (e.g., gender, age, ethnicity), therapy (e.g., referral and assessment dates, treatment information at each appointment) and outcome (improvement, recovery, deterioration) information for individual patients. |
| --- | --- |
| **Hospital Episode Statistics dataset  (NHS Digital, 2021a)** | Admitted Patient Car and Outpatient datasets from all National Health Service (NHS) hospitals across England. Includes demographic (e.g., age, ethnicity, gender), geographical (e.g., residential area, area treatment received), administrative (e.g., dates of admission and discharge), and clinical (e.g., diagnoses, treatments, operations) information for individual patients. |
| **Mental Health Services dataset  (NHS Digital, 2021b)** | Previously known as the Mental Health Minimum Dataset (MHMDS) and the Mental Health and Learning Disability Dataset (MHLDDS). Includes data from secondary care services (e.g., provided in hospitals, outpatient clinics, in the community) for mental illness, learning disability, autism, and other neurodevelopmental conditions. |
| **HES-ONS Mortality dataset  (NHS Digital, 2020)** | Linked information from HES and Office of National Statistics (ONS) mortality data. Includes cause, date, and place of death (both in and out of hospital). |

# NHS Talking Therapies for anxiety and depression Services

*Referral process*: Access to a referral is open to anyone experiencing difficulties with their mental health, regardless of meeting specific thresholds on depression and anxiety symptom scales. People can be referred to NHS TTad by any health or social care professional or can self-refer by contacting their local NHS TTad service. Referral can also be made by a care partner on behalf of the person trying to access therapy. At the point of referral, information is gathered or recorded including contact information, demographics (such as date of birth, gender, ethnicity), and the reason for referral including an option to include diagnoses, and a free-text box for referrers or the person referred to provide any other relevant information. All referrals are triaged by NHS TTad services using the information gathered at referral, at which point the person referred may be placed on an assessment waiting list or invited to book an assessment. They may be referred on to another service if NHS TTad is not the best-placed service to offer the appropriate care, may be discharged back to their General Practitioner (GP), or may be discharged with some sign-posting information.

*Assessment:* NHS TTad services have a target that at least 75% of people referred who go on to access treatment will have waited no more than six weeks between referral and assessment, and 95% will have waited no more than 18 weeks  (National Collaborating Centre for Mental Health, 2025). Assessments take place in person, over the telephone, or occasionally via videoconference. Prior to assessment, people are usually sent electronic self-report questionnaires capturing symptoms of common mental health conditions. During assessments, a practitioner will discuss the questionnaire responses and gather further information to inform treatment decision making. After this assessment, if NHS TTad is appropriate to provide the care needed by the person being referred, then the practitioner will provide various treatment options and may recommend a particular type of therapy. After agreeing the treatment plan, the person referred will be placed on a treatment waiting list or referred on to a more appropriate service. It may also be determined during the assessment that people may not need further care, at which point they are discharged back to their GP or with signposting information.

*Therapy*: If the agreed treatment plan involves care within NHS TTad, people are placed on a waiting list which can range between a few days and several months, after which they will start a course of therapy (National Collaborating Centre for Mental Health, 2025).

Many people will meet diagnostic criteria for several mental health conditions, and our dataset captures information on the condition being treated. People with less complex problems are commonly offered short-term (<=8 sessions) “low intensity” guided self-help therapies, which can be delivered face to face or remotely, in one-to-one or group settings. Where people do not respond to low intensity therapy or their problems are more complex at referral, individuals are commonly offered high intensity therapies (>=16-20 sessions), which are formulation driven one-to-one cognitive behavioural or other types of evidence-based therapies. Interventions are standardized and delivered by trained practitioners and psychotherapists following evidence-based protocols  (National Collaborating Centre for Mental Health, 2021b).

|  | **Condition** | **Psychological therapies** | **Source** |
| --- | --- | --- | --- |
| Step 2: Low-intensity interventions | Depression | Individual guided self-help based on cognitive behavioural therapy (CBT), computerised CBT, behavioural activation, structured group physical activity programme | NICE guidelines NG222, CG91, CG123 |
|  | Generalised anxiety disorder | Self-help, or guided self-help based on CBT, psycho-educational groups, computerised CBT | NICE guidelines CG113, CG123 |
|  | Panic disorder | Self-help, or guided self-help based on CBT, psycho-educational groups, computerised CBT | NICE guidelines CG31, CG123 |
|  | Obsessive-compulsive disorder | Guided self-help based on CBT | NICE guidelines CG31, CG123 |
| Step 3: High intensity interventions | Depression  For individuals with mild to moderate severity who have not responded to initial low-intensity interventions | CBT (individual or group) or interpersonal therapy (IPT)  Behavioural Activation  Couple therapy  Counselling for depression  Brief psychodynamic therapy  Note: Psychological interventions can be provided in combination with antidepressant medication | NICE guidelines: NG222, CG91, CG123 |
|  | Depression  Moderate to severe | CBT (individual) or IPT, each with medication | NICE guidelines: NG222, CG91, CG123 |

Source: National Collaborating Centre for Mental Health. The Improving Access to Psychological Therapies Manual 2021.

# Reasons for discharge

| **Code** | **Description** | **Manuscript discharge category** |
| --- | --- | --- |
|  | **Discharge codes used in NHS TTad from 2012-2014** |  |
| 01 | Completed treatment | Completed |
| 02 | Deceased | Deceased |
| 03 | Declined Treatment | Declined care |
| 04 | Dropped out of treatment (unscheduled discontinuation) | Dropped out |
| 05 | Not suitable for service | Service not suitable |
| 06 | Referral to another service | Referred on |
| 99 | Not Known | Missing / Unknown |
|  | **Discharge codes used in NHS TTad from 2014 onwards** |  |
| 10 | Not suitable for Improving Access to Psychological Therapies Service - no action taken or directed back to referrer | Service not suitable |
| 11 | Not suitable for the Improving Access to Psychological Therapies Service - signposted elsewhere with mutual agreement of PATIENT | Service not suitable |
| 12 | Discharged by mutual agreement following advice and support | Mutual agreement |
| 13 | Referred to another therapy SERVICE by mutual agreement | Referred on |
| 14 | Suitable for Improving Access to Psychological Therapies Service, but PATIENT declined treatment that was offered | Declined care |
| 15 | Deceased (Seen but not taken on for a course of treatment) | Deceased |
| 42 | Completed scheduled treatment | Completed |
| 43 | Dropped out of treatment (unscheduled discontinuation) | Dropped out/mutual discharge |
| 44 | Referred to non IAPT service | Referred on |
| 45 | Deceased | Deceased |
| All other codes assigned to unknown/missing. | | |

# Dementia code list

| **Dementia Subtype** | **ICD-10 code** | **ICD-10 label** | **Associated symptoms** |
| --- | --- | --- | --- |
| **Alzheimer’s disease** | F000 | Dementia in Alzheimer disease with early onset | Cognitive (especially memory) difficulties, mood changes, may include symptoms such as confusion, disorientation, apathy, disturbed sleep, problems with speech, and increasing difficulties with daily living tasks as the disease progresses |
|  | F001 | Dementia in Alzheimer disease with late onset |  |
|  | F009 | Dementia in Alzheimer disease, unspecified |  |
| **Vascular Dementia** | F01 | Vascular dementia | Caused by reduced blood flow to the brain (e.g after a stroke). Early symptoms include problems with concentration, cognitive difficulties, difficulties with walking and balance, and similar difficulties as in Alzheimer’s disease |
| **Mixed or Atypical AD** | F00.2 | Dementia in Alzheimer disease, atypical or mixed type | Alzheimer’s disease dementia with atypical or mixed presentation |
|  | G30.8 | Other Alzheimer disease |  |
| **FTD** | F02.0 | Dementia in Picks disease | Early symptoms may include difficulties with behaviour and language, as well as with memory, planning and organisation, and increasingly physical and movement problems as the disease progresses |
|  | G31.0 | Circumscribed brain atrophy |  |
| **Huntington's disease** | F02.2 | Dementia in Huntington’s disease | Inherited disease-causing brain degeneration. Early symptoms may include cognitive difficulties, mood swings as well as motor difficulties, with increasing motor difficulties as the disease progresses, including with breathing and highly impacting activities of daily living |
|  | G10 | Huntington's disease |  |
| **PD dementia** | F02.3 | Dementia in Parkinson's disease | Early symptoms include problems with movement, followed by cognitive difficulties, memory loss and visuo-spatial problems. |
| **Dementia unspecified** | F03 | Unspecified dementia | Dementia – unspecified subtype. Assignment to this group was only possible if no other dementia code was available in people’s healthcare records. |
| **Other** | F02.4 | Dementia in HIV disease | Codes associated with all-cause dementia in literature, not included as specific subtypes due to sample size or due to lack of specificity of symptoms |
|  | G23.1 | Progressive supranuclear palsy |  |
|  | G31.1 | Senile degeneration of brain, not elsewhere classified Excluded: Alzheimer disease (G30.-) senility NOS (R54) |  |
|  | G31.8 | Other specified degenerative diseases of nervous system Grey-matter degeneration [Alpers] Lewy body(ies)(dementia)(disease) (F02.8*) Subacute necrotizing encephalopathy [Leigh] |  |
|  | E512 | Wernicke encephalopathy |  |
|  | F021 | Dementia in Creutzfeldt-Jakob disease |  |
|  | F028 | Dementia in other specified diseases classified elsewhere |  |
|  | F10.6 | Mental and behavioural disorders due to use of alcohol (Amnesic syndrome) |  |
|  | F10.7 | Mental and behavioural disorders due to use of alcohol (Residual and late-onset psychotic disorder) |  |

# Covariates used in analyses

A range of covariates known to be associated with therapy outcomes (Buckman et al., 2021a, Buckman et al., 2022, Buckman et al., 2021b, Saunders et al., 2021, Saunders et al., 2020) were included in analyses, with the constraint of data availability. Variables were fitted as a categorical variable in the model when a non-linear association with therapy outcomes was found. Categories were defined based on previous research or NHS clinical standards (e.g. age < 65).

| **Variables** | **Identification in databases** | Data source |
| --- | --- | --- |
|  |  |  |
|  |  |  |
|  |  |  |
| **Socio-demographic pre-treatment factors** | | |
| Demographic covariates | Self-reported measures collected at the point of referral: gender (male, female), age, index of multiple deprivation (IMD) quintile (a lowest IMD indicates a higher deprivation area), ethnicity (categories are based on UK census codes ‘White’, ‘Mixed’, ‘Asian’, ‘Black’, and ‘other’) were available in the dataset.  The Index of Multiple Deprivation (IMD) ranks every small area in England from 1 (most deprived area) to 32,844 (least deprived area), based on combined information from seven domains (Income Deprivation; Employment Deprivation, Education; Skills and Training Deprivation; Health Deprivation and Disability; Crime; Barriers to Housing and Services; Living Environment Deprivation.) | NHS TTad |
| Long-term health conditions | During the assessment, all patients are asked whether they have any long-term physical health condition (LTC)l. The type of condition was not available in the dataset. Presence of a long-term condition may be associated with an adaptation in the therapy provided. | NHS TTad |
| Psychotropic Medication taken at start of treatment | Clinicians in the services routinely record whether their patients were prescribed psychotropic medication(s) before starting therapy, this is recorded during the assessment. | NHS TTad |
| Presence of a cardiovascular disease at referral | ICD-10 codes: I20-25, I26-I28, I30-I52, I60-I69 and I70-79 (Huusko et al., 2020) | HES/MHSDS |

Abbreviations: HES=Hospital Episode Statistics, NHS TTad = Improving Access to Psychological Therapies, ICD = International Classification of Disease, MHSDS=Mental Health Services Dataset, N/A = Not applicable, PS = Propensity Score

A measure of NHS Integrated Care Board (ICB) was also included in the propensity score matching algorithm as a categorical variable and in multilevel regressions. Sample size was assessed in each integrated care board, and ICBs with less than 10 people living with dementia were grouped with the closest geographic ICB for analyses purposed.

| **ICB Code** | **Integrated Care Board Name** |
| --- | --- |
| QKK | NHS South East London Integrated Care Board |
| QMF | NHS North East London Integrated Care Board |
| QMJ | NHS North Central London Integrated Care Board |
| QRV | NHS North West London Integrated Care Board |
| QWE | NHS South West London Integrated Care Board |
| QJK | NHS Devon Integrated Care Board |
| QOX | NHS Bath and North East Somerset, Swindon and Wiltshire Integrated Care Board |
| QR1 | NHS Gloucestershire Integrated Care Board |
| QSL | NHS Somerset Integrated Care Board |
| QT6 | NHS Cornwall and The Isles Of Scilly Integrated Care Board |
| QUY | NHS Bristol, North Somerset and South Gloucestershire Integrated Care Board |
| QVV | NHS Dorset Integrated Care Board |
| QKS | NHS Kent and Medway Integrated Care Board |
| QNQ | NHS Frimley Integrated Care Board |
| QNX | NHS Sussex Integrated Care Board |
| QRL | NHS Hampshire and Isle Of Wight Integrated Care Board |
| QU9 | NHS Buckinghamshire, Oxfordshire and Berkshire West Integrated Care Board |
| QXU | NHS Surrey Heartlands Integrated Care Board |
| QGH | NHS Herefordshire and Worcestershire Integrated Care Board |
| QHL | NHS Birmingham and Solihull Integrated Care Board |
| QJ2 | NHS Derby and Derbyshire Integrated Care Board |
| QJM | NHS Lincolnshire Integrated Care Board |
| QK1 | NHS Leicester, Leicestershire and Rutland Integrated Care Board |
| QNC | NHS Staffordshire and Stoke-On-Trent Integrated Care Board |
| QOC | NHS Shropshire, Telford and Wrekin Integrated Care Board |
| QPM | NHS Northamptonshire Integrated Care Board |
| QT1 | NHS Nottingham and Nottinghamshire Integrated Care Board |
| QUA | NHS Black Country Integrated Care Board |
| QWU | NHS Coventry and Warwickshire Integrated Care Board |
| QH8 | NHS Mid and South Essex Integrated Care Board |
| QHG | NHS Bedfordshire, Luton and Milton Keynes Integrated Care Board |
| QJG | NHS Suffolk and North East Essex Integrated Care Board |
| QM7 | NHS Hertfordshire and West Essex Integrated Care Board |
| QMM | NHS Norfolk and Waveney Integrated Care Board |
| QUE | NHS Cambridgeshire and Peterborough Integrated Care Board |
| QE1 | NHS Lancashire and South Cumbria Integrated Care Board |
| QOP | NHS Greater Manchester Integrated Care Board |
| QYG | NHS Cheshire and Merseyside Integrated Care Board |
| QF7 | NHS South Yorkshire Integrated Care Board |
| QHM | NHS North East and North Cumbria Integrated Care Board |
| QOQ | NHS Humber and North Yorkshire Integrated Care Board |
| QWO | NHS West Yorkshire Integrated Care Board |

# Matching Procedure

Propensity-score matching was used to create: 1) a matched “referral” cohort and 2) a matched “assessed” cohort.

To take potential local and service level clustering effect into account, the matching algorithm occurred in two steps. The propensity score was estimated using a logistic regression model, including all-cause dementia (vs no dementia) as the outcome, and all available variables as covariates. To account for potential local service clustering effect, NHS Integrated Care Board was included as a fixed effect in both the propensity score estimation and adjusted for as a covariate in the outcome regression to reduce the risk of bias (Langworthy et al., 2022). Exact matching was also used based on NHS Integrated Care Board, and matching was performed without replacement. Within each NHS ICB, nearest neighbour matching was used, and a calliper set to a maximum of 0.1 for propensity score matching. Matching was performed without replacement. Robust standard errors were used in the matched analyses to account for the fact that the propensity score was estimated.

The quality of the matching was assessed by comparing demographic characteristics of the dementia group before and after matching. A robust variance estimator was used in regressions to account for the fact that the propensity score was estimated.

**Table F1: Referred cohort - Logistic regression model – probability to belong to dementia group, and demographic characteristics in the matched cohort**

| **N=4 832 112** | **Coefficient** | **Standard error** | **p-value** | **Characteristics after matching** | |  |
| --- | --- | --- | --- | --- | --- | --- |
|  |  |  |  | **Dementia (N=6623)** | **No Dementia (N=6623)** |  |
| **Age Group** – 18-24 | ref |  |  | 121 | 116 |  |
| 25-44 | 0.67 | 0.099 | <.0001 | 632 | 632 |  |
| 45-64 | 1.97 | 0.094 | <.0001 | 1688 | 1691 |  |
| 65+ | 3.98 | 0.094 | <.0001 | 4182 | 4184 |  |
| **IMD quintile** – 1st | ref |  |  | 1652 | 1652 |  |
| 2 | -0.20 | 0.038 | <.0001 | 1384 | 1390 |  |
| 3 | -0.23 | 0.389 | <.0001 | 1297 | 1301 |  |
| 4 | -0.36 | 0.041 | <.0001 | 1080 | 1072 |  |
| 5 | -0.43 | 0.044 | <.0001 | 956 | 963 |  |
| Missing | -0.29 | 0.069 | <.0001 | 254 | 245 |  |
| **CV disease at referral** | 1.60 | 0.027 | <.0001 | 3049 | 3042 |  |
| **Gender** (ref male) | ref |  |  | 2746 | 2748 |  |
| Female | -0.14 | 0.026 | <.0001 | 3778 | 3773 |  |
| Missing | 0.10 | 0.107 | 0.3584 | 99 | 102 |  |
| **Ethnicity** – White British | Ref |  |  | 3937 | 3947 |  |
| White Irish | 0.22 | 0.107 | 0.0395 | 94 | 93 |  |
| Other White Background | -0.13 | 0.088 | 0.1266 | 141 | 141 |  |
| White and Black Caribbean | 0.33 | 0.211 | 0.1176 | 23 | 20 |  |
| White and Black African | -0.38 | 0.579 | 0.5136 | 3 | 3 |  |
| White and Asian | 0.49 | 0.280 | 0.0795 | 13 | 6 |  |
| Other mixed background | -0.22 | 0.226 | 0.3125 | 20 | 18 |  |
| Indian | 0.19 | 0.089 | 0.0336 | 146 | 145 |  |
| Pakistani | 0.37 | 0.125 | 0.0030 | 68 | 70 |  |
| Bangladeshi | 0.23 | 0.241 | 0.3435 | 18 | 15 |  |
| Other Asian Background | 0.03 | 0.145 | 0.8436 | 50 | 46 |  |
| Caribbean | 0.37 | 0.104 | 0.005 | 102 | 97 |  |
| African | 0.17 | 0.158 | 0.2717 | 42 | 37 |  |
| Other Black Background | 0.64 | 0.173 | 0.002 | 35 | 29 |  |
| Chinese | -0.87 | 0.579 | 0.1354 | ND | ND |  |
| Other ethnic group | 0.05 | 0.133 | 0.7280 | 60 | 63 |  |
| Missing | 0.36 | 0.362 | <.0001 | 1868 | 1889 |  |
| **NHS ICB** – Mid and South Essex | Ref |  |  | 105 | 105 |  |
| Lancashire and South Cumbria | 0.20 | 0.120 | 0.0952 | 213 | 213 |  |
| South Yorkshire | -0.26 | 0.133 | 0.0514 | 127 | 127 |  |
| Bedfordshire, Luton and Milton Keynes | 0.21  210.63 | 0.146 | 0.1576 | 87 | 87 |  |
| Birmingham and Solihull | 0.63 | 0.124 | <.0001 | 186 | 186 |  |
| Herefordshire and Worcestershire | -0.30 | 0.115 | 0.0097 | 286 | 286 |  |
| Derby and Derbyshire | 0.28 | 0.135 | 0.0373 | 104 | 104 |  |
| Suffolk and North East Essex | -0.31 | 0.140 | 0.0276 | 118 | 118 |  |
| Devon | -0.17 | 0.167 | 0.3148 | 100 | 100 |  |
| Lincolnshire | -0.27 | 0.168 | 0.1139 | 55 | 55 |  |
| Leicester, Leicestershire and Rutland | 0.26 | 0.122 | 0.0337 | 54 | 54 |  |
| South East London | 0.06 | 0.126 | 0.6483 | 200 | 200 |  |
| Kent and Medway | -0.06 | 0.137 | 0.6500 | 162 | 162 |  |
| Hertfordshire and West Essex | 0.40 | 0.122 | 0.0010 | 110 | 110 |  |
| North East London | 0.48 | 0.118 | <.0001 | 211 | 211 |  |
| North Central London | -0.22 | 0.143 | 0.1312 | 252 | 252 |  |
| Norfolk and Waveney | 0.03  -00 | 0.143 | 0.8480 | 92 | 92 |  |
| Staffordshire and Stoke-On-Trent | -0.09 | 0.167 | 0.5933 | 93 | 93 |  |
| Frimley | -0.47 | 0.137 | 0.0006 | 56 | 56 |  |
| Sussex | 0.27 | 0.174 | 0.1166 | 109 | 109 |  |
| Shropshire, Telford and Wrekin | 0.26 | 0.110 | 0.0169 | 470 | 470 |  |
| Greater Manchester | -0.29 | 0.137 | 0.0338 | 110 | 110 |  |
| Humber and North Yorkshire | -0.21 | 0.145 | 0.1540 | 89 | 89 |  |
| Bath and North East Somerset, Swindon and Wiltshire | -0.02 | 0.168 | 0.9112 | 54 | 54 |  |
| Northamptonshire | 0.31 | 0.163 | 0.0575 | 60 | 60 |  |
| Gloucestershire | 0.69 | 0.118 | <.0001 | 236 | 236 |  |
| Hampshire and Isle of Wight | 1.24 | 0.106 | <.0001 | 771 | 771 |  |
| North West London | -0.20 | 0.166 | 0.2190 | 57 | 57 |  |
| Somerset | -0.014 | 0.143 | 0.9218 | 93 | 93 |  |
| Nottingham and Nottinghamshire | 0.12 | 0.143 | 0.4216 | 97 | 94 |  |
| Cornwall and The Isles of Scilly | -0.11 | 0.132 | 0.4103 | 131 | 131 |  |
| Buckinghamshire, Oxfordshire and Berkshire West | 0.20 | 0.139 | 0.1146 | 147 | 147 |  |
| Black Country | 0.67 | 0.139 | <.0001 | 106 | 106 |  |
| Cambridgeshire and Peterborough | 0.35 | 0.143 | 0.0155 | 94 | 94 |  |
| Bristol, North Somerset and South Gloucestershire | -0.14 | 0.150 | 0.3469 | 78 | 78 |  |
| Dorset | 0.29 | 0.128 | 0.0231 | 150 | 150 |  |
| South West London | -0.11 | 0.125 | 0.3991 | 169 | 169 |  |
| West Yorkshire | -0.09 | 0.160 | 0.5908 | 63 | 63 |  |
| Coventry and Warwickshire | 0.44 | 0.144 | 0.0022 | 93 | 93 |  |
| Surrey Heartlands | 0.95 | 0.106 | <.0001 | 714 | 714 |  |
| Missing | 0.19 | 0.152 | 0.2169 | 75 | 75 |  |
| Intercept | -8.98 | 0.137 | <.0001 | - | - |  |
| Abbreviations: IMD = Index of Multiple Deprivation, ICB = Integrated care board, CV = cardiovascular, ND = Not disclosed for confidentiality reasons due to small cell size (<=5)  Note: The most granular ethnicity variable was included in the matching procedure. Categories were combined in further analyses to allow for adequate sample size in each category. | | | | | | |

**Table F2: Assessed cohort - Logistic regression model – probability to belong to dementia group, and demographic characteristics in the matched cohort**

| **N=3 194 962** | **Coefficient** | **Standard error** | **p-value** | **Characteristics after matching** | |  |
| --- | --- | --- | --- | --- | --- | --- |
|  |  |  |  | **Dementia (N=3997)** | **No Dementia (N=3997)** |  |
| **Age Group** - 18-24 | ref |  |  | 60 | 52 |  |
| 25-44 | 0.64 | 0.140 | <.0001 | 353 | 348 |  |
| 45-64 | 1.83 | 0.133 | <.0001 | 1067 | 1085 |  |
| 65+ | 3.70 | 0.133 | <.0001 | 2517 | 2512 |  |
| **IMD quintile** – 1st | ref |  |  | 989 | 992 |  |
| 2 | -0.23 | 0.048 | <.0001 | 852 | 840 |  |
| 3 | -0.30 | 0.050 | <.0001 | 774 | 765 |  |
| 4 | -0.41 | 0.053 | <.0001 | 660 | 668 |  |
| 5 | -0.50 | 0.57 | <.0001 | 559 | 570 |  |
| Missing | -0.20 | 0.087 | 0.0227 | 163 | 162 |  |
| **CV disease at referral** (Yes vs No) | 1.51 | 0.036 | <.0001 | 1864 | 1867 |  |
| **Gender** = Male | Ref |  |  | 1722 | 1730 |  |
| Female | -0.18 | 0.033 | <.0001 | 2222 | 2220 |  |
| Missing | 0.30 | 0.148 | 0.0398 | 53 | 47 |  |
| Taking psychotropic medication at referral (ref=No) |  |  |  | 1285 | 1285 |  |
| Yes | 0.40 | 0.037 | <.0001 | 1758 | 1773 |  |
| Missing | 0.71 | 0.047 | <.0001 | 954 | 939 |  |
| Self-reported long-term health condition (ref=No) |  |  |  | 905 | 903 |  |
| Yes | 0.60 | 0.043 | <.0001 | 1863 | 1878 |  |
| Missing | 0.43 | 0.049 | <.0001 | 1229 | 1216 |  |
| **Ethnicity** – White British | Ref |  |  | 2734 | 2785 |  |
| White Irish | 0.15 | 0.127 | 0.2270 | 67 | 65 |  |
| Other White Background | -0.13 | 0.103 | 0.2031 | 104 | 100 |  |
| White and Black Caribbean | 0.36 | 0.246 | 0.1424 | 17 | 17 |  |
| White and Black African | -0.47 | 0.709 | 0.5108 | 2 | 2 |  |
| White and Asian | 0.34 | 0.357 | 0.3403 | 8 | 5 |  |
| Other mixed background | -0.13 | 0.261 | 0.6184 | 15 | 9 |  |
| Indian | 0.06 | 0.108 | 0.5640 | 99 | 93 |  |
| Pakistani | 0.257 | 0.148 | 0.0822 | 49 | 44 |  |
| Bangladeshi | 0.140 | 0.274 | 0.6099 | 14 | 13 |  |
| Other Asian Background | -0.102 | 0.177 | 0.5636 | 34 | 28 |  |
| Caribbean | 0.358 | 0.121 | 0.0030 | 77 | 80 |  |
| African | 0.140 | 0.185 | 0.4468 | 31 | 20 |  |
| Other Black Background | 0.811 | 0.189 | <.0001 | 30 | 21 |  |
| Chinese | -0.860 | 0.709 | 0.2256 | ND | ND |  |
| Other ethnic group | 0.038 | 0.155 | 0.8075 | 44 | 39 |  |
| Missing | 0.068 | 0.050 | 0.1764 | 670 | 671 |  |
| **NHS ICB** – Mid and South Essex | ref |  |  | 78 | 78 |  |
| Lancashire and South Cumbria | 0.23 | 0.141 | 0.1064 | 151 | 151 |  |
| South Yorkshire | -0.26 | 0.167 | 0.1250 | 69 | 69 |  |
| Bedfordshire, Luton and Milton Keynes | 0.37 | 0.175 | 0.0335 | 59 | 59 |  |
| Birmingham and Solihull | 0.86 | 0.142 | <.0001 | 152 | 152 |  |
| Herefordshire and Worcestershire | -0.28 | 0.137 | 0.0406 | 182 | 182 |  |
| North East and North Cumbria | -0.09 | 0.178 | 0.6089 | 55 | 55 |  |
| Derby and Derbyshire | 0.305 | 0.158 | 0.0529 | 87 | 87 |  |
| Suffolk and North East Essex | -0.22 | 0.169 | 0.1958 | 67 | 67 |  |
| Devon | -0.26 | 0.225 | 0.2542 | 27 | 27 |  |
| Lincolnshire | -0.26 | 0.214 | 0.2246 | 31 | 31 |  |
| Leicester, Leicestershire and Rutland | 0.30 | 0.148 | 0.0445 | 120 | 120 |  |
| South East London | 0.16 | 0.156 | 0.3117 | 92 | 92 |  |
| Kent and Medway | 0.01 | 0.163 | 0.9560 | 76 | 76 |  |
| Hertfordshire and West Essex | 0.62 | 0.140 | <.0001 | 171 | 171 |  |
| North East London | 0.40 | 0.146 | 0.0062 | 130 | 130 |  |
| North Central London | -0.19 | 0.170 | 0.2712 | 64 | 64 |  |
| Norfolk and Waveney | -0.03 | 0.179 | 0.8540 | 54 | 54 |  |
| Staffordshire and Stoke-On-Trent | 0.12 | 0.198 | 0.5383 | 39 | 39 |  |
| Frimley | -0.43 | 0.170 | 0.0114 | 65 | 65 |  |
| Sussex | 0.55 | 0.200 | 0.0059 | 38 | 38 |  |
| Shropshire, Telford and Wrekin | 0.30 | 0.129 | 0.0194 | 308 | 308 |  |
| Greater Manchester | -0.29 | 0.178 | 0.1098 | 54 | 54 |  |
| Humber and North Yorkshire | -0.06 | 0.160 | 0.7283 | 82 | 82 |  |
| Bath and North East Somerset, Swindon and Wiltshire | -0.05 | 0.225 | 0.8132 | 27 | 27 |  |
| Northamptonshire | 0.17 | 0.199 | 0.4066 | 38 | 38 |  |
| Gloucestershire | 0.52 | 0.145 | 0.0003 | 128 | 128 |  |
| Hampshire and Isle of Wight | 1.37 | 0.126 | <.0001 | 483 | 483 |  |
| North West London | 0.12 | 0.200 | 0.5443 | 38 | 38 |  |
| Somerset | 0.14 | 0.167 | 0.3999 | 69 | 69 |  |
| Nottingham and Nottinghamshire | 0.26 | 0.170 | 0.1248 | 66 | 66 |  |
| Cornwall and The Isles of Scilly | -0.36 | 0.167 | 0.0318 | 70 | 70 |  |
| Buckinghamshire, Oxfordshire and Berkshire West | 0.39 | 0.151 | 0.0095 | 109 | 109 |  |
| Black Country | 0.66 | 0.162 | <.0001 | 78 | 78 |  |
| Cambridgeshire and Peterborough | 0.38 | 0.167 | 0.0215 | 69 | 69 |  |
| Bristol, North Somerset and South Gloucestershire | -0.25 | 0.192 | 0.2020 | 43 | 43 |  |
| Dorset | 0.39 | 0.158 | 0.0136 | 87 | 87 |  |
| South West London | -0.18 | 0.151 | 0.2379 | 102 | 102 |  |
| West Yorkshire | 0.16 | 0.189 | 0.4009 | 45 | 45 |  |
| Coventry and Warwickshire | 0.79 | 0.167 | <.0001 | 71 | 71 |  |
| Surrey Heartlands | 0.12 | 0.137 | 0.3634 | 181 | 181 |  |
| Missing | 0.29 | 0.193 | 0.1557 | 42 | 42 |  |
| Intercept | -9.46 | 0.179 | <.0001 | N/A | N/A |  |
| IMD = Index of Multiple Deprivation, ICB = Integrated care board, CV = cardiovascular, ND = Not disclosed for confidentiality reasons due to small cell size (<=5)  Note: N/A=Not applicable. The most granular ethnicity variable was included in the matching procedure. Categories were combined in further analyses to allow for adequate sample size in each category. | | | | | | |

# Supplementary analyses - Dementia Subtypes disparities

To better understand disparities by subtype within the dementia population, and whether some of the less common forms of dementia are associated with different treatment pathways, supplementary analyses were also conducted to examine whether subtypes of dementia are associated with different treatment pathways, and whether this differs by age group, with the caveat that such analyses may have less statistical power due to the lower prevalence of non-memory led dementia presentations, such as frontotemporal dementia.

The following analyses were conducted:

1. Multilevel logistic regressions were used to evaluate whether treatment pathways differed according to each dementia subtype. Because people may be diagnosed with several dementia subtypes, Model 4 (as above) was re-run sequentially including each subtype of dementia (vs all other subtypes) as a covariate. An additional model was run to evaluate whether being diagnosed with several types of dementia is associated with different treatment pathways. (Supplementary Table G1)
2. To reflect the varying nature of healthcare services provided for “older adults” (>=65 years old) vs younger adults in the NHS, access rates were also calculated by age category for each subtype of dementia. (Supplementary Table G2)

**Table G1 Demographic characteristics and outcomes by subtype of dementia**

| **Dementia Type** | **N Referred** | **Mean Age (SD)** | **Gender - % female** | **Ethnicity - % minority group** | **Outcome: % Received an assessment** | | | **Outcome: % Received therapy** | | |
| --- | --- | --- | --- | --- | --- | --- | --- | --- | --- | --- |
|  |  |  |  |  | **%** | **OR (95% CI)** | **p-value** | **Received treatment %** | **OR (95% CI)** | **p-value** |
| All cause Dementia - ref | 6623 |  |  |  |  |  |  |  |  |  |
| AD | 2193 | 70.5 (16.5) | 60.2 | 12.5 | 55.0 | 0.75 (0.66; 0.84) | <.0001 | 43.0 | 0.95 (0.82; 1.11) | 0.5077 |
| VaD | 1628 | 70.9 (16.0) | 54.8 | 10.8 | 60.0 | 0.98 (0.85; 1.12) | 0.7266 | 43.6 | 1.01 (0.86; 1.20) | 0.8788 |
| Atypical or mixed AD | 873 | 70.6 (18.0) | 61.3 | 11.8 | 53.3 | 0.73 (0.62; 0.87) | 0.0003 | 42.6 | 1.03 (0.83; 1.27) | 0.8201 |
| Huntington’s | 364 | 44.7 (15.8) | 55.1 | 4.8 | 52.7 | 0.73 (0.56; 0.95) | 0.0212 | 47.9 | 1.00 (0.71; 1.40) | 0.9945 |
| FTD | 280 | 55.5 (17.2) | 51.4 | 7.9 | 46.1 | 0.48 (0.35; 0.65) | <.0001 | 44.2 | 0.90 (0.60; 1.36) | 0.6324 |
| PD dementia | 348 | 57.8 (20.6) | 47.6 | 9.02 | 54.6 | 0.71 (0.55; 0.93) | 0.0133 | 40.0 | 0.79 (0.57; 1.10) | 0.1646 |
| Mixed presentations | 502 | 64.5 (20.3) | 49.6 | 7.6 | 54.0 | 0.72 (0.58; 0.90) | 0.0033 | 42.4 | 0.99 (0.75; 1.31) | 0.9427 |
| Other | 1488 | 54.3 (15.8) | 51.3 | 8.1 | 64.0 | 1.16 (1.00; 1.34) | 0.0542 | 47.0 | 0.94 (0.79; 1.11) | 0.4748 |
| Dementia unspecified | 1369 | 67.5 (16.2) | 63 | 17.1 | 62.9 | 1.15 (1.00; 1.32) | 0.0490 | 47.0 | 1.06 (0.90; 1.25) | 0.4580 |
| Abbreviations: AD = Alzheimer’s disease, VaD = Vascular Dementia, FTD=Frontotemporal Dementia, PD = Parkinson’s Disease, OR = Odds Ratio, CI = Confidence Interval  Note: One multilevel logistic regression model was run for each subtype separately in the all-cause dementia cohort, including dementia subtype (subtype vs all others), age, gender, ethnicity (white vs other ethnicities), Index of Multiple Deprivation quintile, presence of cardiovascular disease before referral as fixed effects, and NHS integrated care board as a random intercept.  Models using “receiving treatment” as an outcome were additionally adjusted for taking psychotropic medications, presence of a long-term health condition and depression and anxiety symptom severity. Percentages for gender and ethnicity are calculated out of non-missing data. | | | | | | | | | | |

**Table G2 Outcomes by age group and subtype**

| **Subtype**  **Reason for discharge** | **REFERRAL COHORT** | | | | **ASSESSED COHORT** | | | |
| --- | --- | --- | --- | --- | --- | --- | --- | --- |
|  | **Age < 65**  **N (%)** | | **Age >= 65**  **N (%)** | | **Age < 65**  **N (%)** | | **Age >= 65**  **N (%)** | |
| **AD** |  |  |  |  |  |  |  |  |
| **Assessed** | **269** | **50.3%** | **938** | **56.6%** | **123** | **45.7%** | **396** | **42.2%** |
| **Dropout** | **23** | **4.3%** | **35** | **2.1%** | **16** | **5.9%** | **50** | **5.3%** |
| **Service NS** | **111** | **20.7%** | **293** | **17.7%** | **40** | **14.9%** | **99** | **10.6%** |
| **Declined care** | **54** | **10.1%** | **128** | **7.7%** | **11** | **4.1%** | **43** | **4.6%** |
| **Referred on** | **20** | **3.7%** | **100** | **6.0%** | **38** | **14.1%** | **88** | **9.4%** |
| **Mutual agreement** | **12** | **2.2%** | **31** | **1.9%** | **6** | **2.2%** | **39** | **4.2%** |
| **Deceased** | **0** | **0.0%** | **3** | **0.2%** | **2** | **0.7%** | **3** | **0.3%** |
| **Ongoing** | **0** | **0.0%** | **0** | **0.0%** | **0** | **0.0%** | **0** | **0.0%** |
| **Missing** | **46** | **8.6%** | **130** | **7.8%** | **33** | **12.3%** | **220** | **23.5%** |
|  | **535** |  | **1658** |  | **269** |  | **938** |  |
| **VaD** |  |  |  |  |  |  |  |  |
| **Assessed** | **204** | **52.8%** | **773** | **62.2%** | **89** | **43.4%** | **337** | **42.2%** |
| **Dropout** | **17** | **4.4%** | **22** | **1.8%** | **10** | **4.9%** | **74** | **9.3%** |
| **Service NS** | **74** | **19.2%** | **192** | **15.5%** | **37** | **18.0%** | **80** | **10.0%** |
| **Declined care** | **44** | **11.4%** | **87** | **7.0%** | **10** | **4.9%** | **49** | **6.1%** |
| **Referred on** | **17** | **4.4%** | **69** | **5.6%** | **30** | **14.6%** | **65** | **8.1%** |
| **Mutual agreement** | **4** | **1.0%** | **19** | **1.5%** | **1** | **0.5%** | **26** | **3.3%** |
| **Deceased** | **0** | **0.0%** | **0** | **0.0%** | **2** | **1.0%** | **1** | **0.1%** |
| **Ongoing** | **0** | **0.0%** | **0** | **0.0%** | **0** | **0.0%** | **0** | **0.0%** |
| **Missing** | **26** | **6.7%** | **80** | **6.4%** | **26** | **12.7%** | **167** | **20.9%** |
|  | **386** |  | **1242** |  | **205** |  | **799** |  |
| **Atypical or mixed AD** |  |  |  |  |  |  |  |  |
| **Assessed** | **83** | **43.9%** | **382** | **55.8%** | **31** | **37.3%** | **167** | **43.7%** |
| **Dropout** | **8** | **4.2%** | **14** | **2.0%** | **5** | **6.0%** | **17** | **4.5%** |
| **Service NS** | **56** | **29.6%** | **122** | **17.8%** | **20** | **24.1%** | **36** | **9.4%** |
| **Declined care** | **22** | **11.6%** | **43** | **6.3%** | **4** | **4.8%** | **18** | **4.7%** |
| **Referred on** | **7** | **3.7%** | **52** | **7.6%** | **15** | **18.1%** | **39** | **10.2%** |
| **Mutual agreement** | **3** | **1.6%** | **9** | **1.3%** | **1** | **1.2%** | **16** | **4.2%** |
| **Deceased** |  | **0.0%** |  | **0.0%** | **1** | **1.2%** | **1** | **0.3%** |
| **Ongoing** |  | **0.0%** |  | **0.0%** | **2** | **2.4%** | **9** | **2.4%** |
| **Missing** | **10** | **5.3%** | **62** | **9.1%** | **4** | **4.8%** | **79** | **20.7%** |
|  | **189** |  | **684** |  | **83** |  | **382** |  |
| **Huntington's** |  |  |  |  |  |  |  |  |
| **Assessed** | **164** | **50.5%** | **28** | **71.8%** | **75** | **45.7%** | **17** | **60.7%** |
| **Dropout** | **15** | **4.6%** | **0** | **0.0%** | **8** | **4.9%** | **1** | **3.6%** |
| **Service NS** | **70** | **21.5%** | **4** | **10.3%** | **25** | **15.2%** | **5** | **17.9%** |
| **Declined care** | **44** | **13.5%** | **3** | **7.7%** | **8** | **4.9%** | **4** | **14.3%** |
| **Referred on** | **12** | **3.7%** | **1** | **2.6%** | **27** | **16.5%** | **0** | **0.0%** |
| **Mutual agreement** | **1** | **0.3%** | **1** | **2.6%** | **2** | **1.2%** | **0** | **0.0%** |
| **Deceased** |  | **0.0%** |  | **0.0%** | **1** | **0.6%** | **0** | **0.0%** |
| **Ongoing** |  | **0.0%** |  | **0.0%** | **4** | **2.4%** | **1** | **3.6%** |
| **Missing** | **19** | **5.8%** | **2** | **5.1%** | **14** | **8.5%** | **0** | **0.0%** |
|  | **325** |  | **39** |  | **164** |  | **28** |  |
| **FTD** |  |  |  |  |  |  |  |  |
| **Assessed** | **89** | **44.3%** | **40** | **50.6%** | **37** | **41.6%** | **20** | **50.0%** |
| **Dropout** | **9** | **4.5%** | **0** | **0.0%** | **4** | **4.5%** | **1** | **2.5%** |
| **Service NS** | **56** | **27.9%** | **18** | **22.8%** | **20** | **22.5%** | **4** | **10.0%** |
| **Declined care** | **25** | **12.4%** | **7** | **8.9%** | **4** | **4.5%** | **6** | **15.0%** |
| **Referred on** | **10** | **5.0%** | **6** | **7.6%** | **13** | **14.6%** | **5** | **12.5%** |
| **Mutual agreement** | **2** | **1.0%** | **2** | **2.5%** | **1** | **1.1%** | **0** | **0.0%** |
| **Deceased** |  | **0.0%** |  | **0.0%** | **2** | **2.2%** | **1** | **2.5%** |
| **Ongoing** |  | **0.0%** |  | **0.0%** | **1** | **1.1%** | **0** | **0.0%** |
| **Missing** | **10** | **5.0%** | **6** | **7.6%** | **7** | **7.9%** | **3** | **7.5%** |
|  | **201** |  | **79** |  | **89** |  | **40** |  |
| **PD Dementia** |  |  |  |  |  |  |  |  |
| **Assessed** | **71** | **42.0%** | **119** | **66.5%** | **25** | **35.2%** | **51** | **42.9%** |
| **Dropout** | **7** | **4.1%** | **3** | **1.7%** | **4** | **5.6%** | **7** | **5.9%** |
| **Service NS** | **53** | **31.4%** | **27** | **15.1%** | **18** | **25.4%** | **14** | **11.8%** |
| **Declined care** | **19** | **11.2%** | **12** | **6.7%** | **6** | **8.5%** | **7** | **5.9%** |
| **Referred on** | **8** | **4.7%** | **7** | **3.9%** | **10** | **14.1%** | **12** | **10.1%** |
| **Mutual agreement** | **2** | **1.2%** | **2** | **1.1%** | **0** | **0.0%** | **0** | **0.0%** |
| **Deceased** |  | **0.0%** |  | **0.0%** | **1** | **1.4%** | **0** | **0.0%** |
| **Ongoing** |  | **0.0%** |  | **0.0%** | **2** | **2.8%** | **2** | **1.7%** |
| **Missing** | **9** | **5.3%** | **9** | **5.0%** | **5** | **7.0%** | **26** | **21.8%** |
|  | **169** |  | **179** |  | **71** |  | **119** |  |
| **Mixed presentations** |  |  |  |  |  |  |  |  |
| **Assessed** | **77** | **43.5%** | **194** | **59.7%** | **30** | **39.0%** | **85** | **43.8%** |
| **Dropout** | **6** | **3.4%** | **3** | **0.9%** | **3** | **3.9%** | **8** | **4.1%** |
| **Service NS** | **55** | **31.1%** | **56** | **17.2%** | **21** | **27.3%** | **22** | **11.3%** |
| **Declined care** | **20** | **11.3%** | **21** | **6.5%** | **4** | **5.2%** | **11** | **5.7%** |
| **Referred on** | **10** | **5.6%** | **20** | **6.2%** | **12** | **15.6%** | **20** | **10.3%** |
| **Mutual agreement** | **2** | **1.1%** | **4** | **1.2%** | **0** | **0.0%** | **4** | **2.1%** |
| **Deceased** | **0** | **0.0%** | **0** | **0.0%** | **1** | **1.3%** | **1** | **0.5%** |
| **Ongoing** | **0** | **0.0%** | **0** | **0.0%** | **0** | **0.0%** | **0** | **0.0%** |
| **Missing** | **7** | **4.0%** | **27** | **8.3%** | **6** | **7.8%** | **43** | **22.2%** |
|  | **177** |  | **325** |  | **77** |  | **194** |  |
| **Other** |  |  |  |  |  |  |  |  |
| **Assessed** | **678** | **62.3%** | **274** | **68.5%** | **327** | **48.2%** | **120** | **38.1%** |
| **Dropout** | **41** | **3.8%** | **10** | **2.5%** | **50** | **7.4%** | **13** | **4.1%** |
| **Service NS** | **143** | **13.1%** | **40** | **10.0%** | **80** | **11.8%** | **26** | **8.3%** |
| **Declined care** | **109** | **10.0%** | **32** | **8.0%** | **24** | **3.5%** | **17** | **5.4%** |
| **Referred on** | **31** | **2.8%** | **14** | **3.5%** | **75** | **11.1%** | **35** | **11.1%** |
| **Mutual agreement** | **14** | **1.3%** | **4** | **1.0%** | **12** | **1.8%** | **5** | **1.6%** |
| **Deceased** | **1** | **0.1%** | **0** | **0.0%** | **5** | **0.7%** | **2** | **0.6%** |
| **Ongoing** | **0** | **0.0%** | **0** | **0.0%** | **0** | **0.0%** | **56** | **17.8%** |
| **Missing** | **71** | **6.5%** | **26** | **6.5%** | **105** | **15.5%** | **41** | **13.0%** |
|  | **1088** |  | **400** |  | **678** |  | **315** |  |
| **Dementia unspecified** |  |  |  |  |  |  |  |  |
| **Assessed** | **328** | **63.0%** | **533** | **62.9%** | **176** | **53.7%** | **229** | **43.0%** |
| **Dropout** | **26** | **5.0%** | **21** | **2.5%** | **23** | **7.0%** | **37** | **6.9%** |
| **Service NS** | **49** | **9.4%** | **86** | **10.1%** | **23** | **7.0%** | **50** | **9.4%** |
| **Declined care** | **55** | **10.6%** | **69** | **8.1%** | **18** | **5.5%** | **34** | **6.4%** |
| **Referred on** | **16** | **3.1%** | **46** | **5.4%** | **40** | **12.2%** | **49** | **9.2%** |
| **Mutual agreement** | **4** | **0.8%** | **8** | **0.9%** | **6** | **1.8%** | **16** | **3.0%** |
| **Deceased** | **1** | **0.2%** | **0** | **0.0%** | **2** | **0.6%** | **10** | **1.9%** |
| **Ongoing** | **0** | **0.0%** | **1** | **0.1%** | **10** | **3.0%** | **13** | **2.4%** |
| **Missing** | **42** | **8.1%** | **84** | **9.9%** | **30** | **9.1%** | **95** | **17.8%** |
|  | **521** |  | **848** |  | **328** |  | **533** |  |

# Expected prevalence of dementia in general depression population over 35

|  | **Prevalence (%)** | **Reference/calculation** |
| --- | --- | --- |
| Estimated point prevalence of depression in mild-to-moderate dementia: | 38.0% | Leung 2021 |
| Estimated prevalence of mild-to-moderate dementia in over 35 years old | 1.3% | Wittenberg 2019 |
| Estimated point-prevalence of depression + mild-to-moderate dementia in general population | 0.494% | = 38 * 1.3 / 100 |
| Estimated point prevalence of depression in the general population | 12.9% | Lim 2018 |
| Estimated point-prevalence of mild-to-moderate dementia in general depression population | 3.82% | = 0.494*100/12.9 |

# Local disparities

To illustrate these local variabilities geographically, access rates for people with dementia are presented by region in Figure G1. Rates of access to assessment varied from 55.69% in Yorkshire to 76.94% in the West Midlands, and rates of access to treatment varied from 31.13% in the North West of England to 57.63% in the East Midlands.

**Figure I1 Rates of access to assessment and treatment by region in England**

**% Assessed % received therapy**


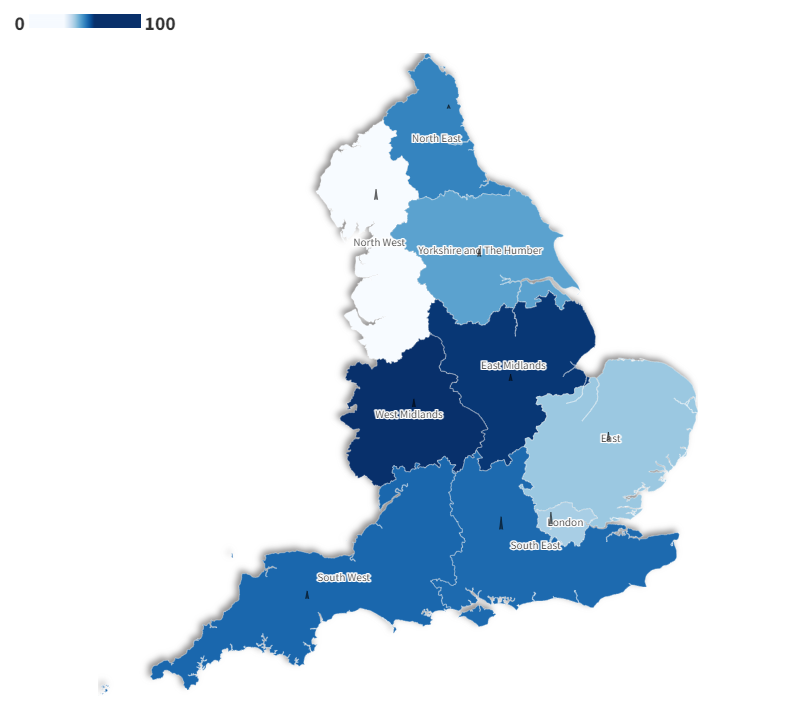

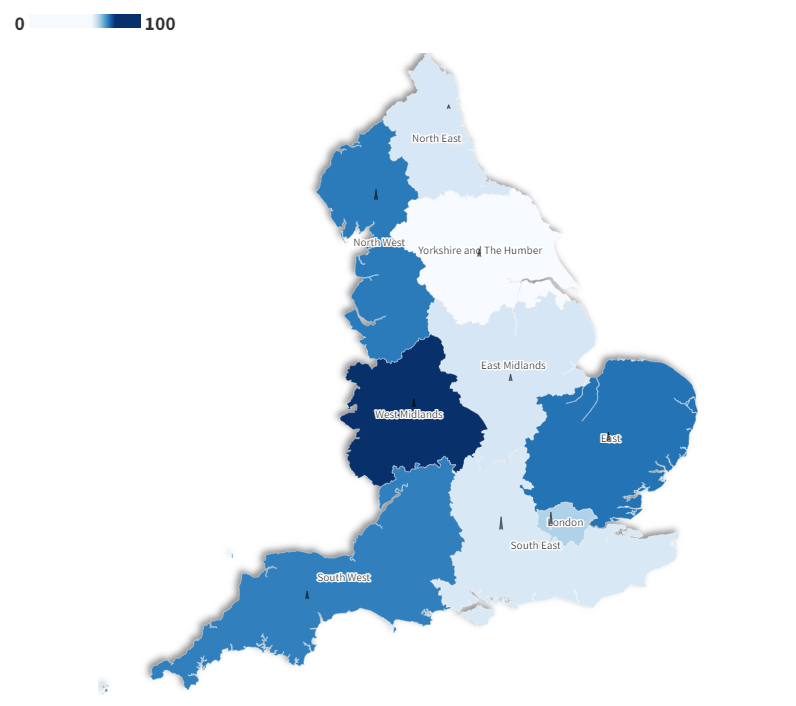


Note: Maps created with Flourish Studio

# Authors contributions

**Author contributions**: C.E.B., E.B, A.J., J.S., R.S., M.R. C.C. were involved in the conceptualisation and design of the study. C.E.B., E.B, A.J., J.S., R.S. and J.E.J.B. contributed to the methodology. E.B, S.C and J.S were involved in the atypical dementia ascertainment consensus meeting. A.J, R.S and C.E.B were involved in data extraction and acquisition. C.E.B. carried out the analyses. C.E.B, A.J. and R.S. assessed and verified the underlying data reported in the manuscript. N.M and V.M were PPI representative. C.E.B wrote the first draft of the manuscript, all authors contributed to the writing and review of subsequent versions of the manuscript and approved the final version. All authors had full access to all data in the study and accept the responsibility to submit for publication.

# The RECORD statement – checklist of items, applied to the present study

|  | **Item No.** | **STROBE items** | **Location in manuscript where items are reported** | **RECORD items** | **Location in manuscript where items are reported** |  |
| --- | --- | --- | --- | --- | --- | --- |
| **Title and abstract** | | | | | | |
|  | 1 | (a) Indicate the study’s design with a commonly used term in the title or the abstract (b) Provide in the abstract an informative and balanced summary of what was done and what was found | (a) Title/Abstract (b) Abstract | RECORD 1.1: The type of data used should be specified in the title or abstract. When possible, the name of the databases used should be included.  RECORD 1.2: If applicable, the geographic region and timeframe within which the study took place should be reported in the title or abstract.  RECORD 1.3: If linkage between databases was conducted for the study, this should be clearly stated in the title or abstract. | Title, Methods -Databases  Title, Methods - Databases, Abstract  Title |  |
| **Introduction** | | | | | | |
| Background rationale | 2 | Explain the scientific background and rationale for the investigation being reported | Introduction |  | Introduction |  |
| Objectives | 3 | State specific objectives, including any prespecified hypotheses | Research aims |  | Research aims |  |
| **Methods** | | | | | | |
| Study Design | 4 | Present key elements of study design early in the paper | Methods – Databases and procedures |  | Methods – Databases and procedures |  |
| Setting | 5 | Describe the setting, locations, and relevant dates, including periods of recruitment, exposure, follow-up, and data collection | Methods – Study population |  |  |  |
| Participants | 6 | *(a) Case-control study* - Give the eligibility criteria, and the sources and methods of case ascertainment and control selection. Give the rationale for the choice of cases and controls  *(b) Case-control study* - For matched studies, give matching criteria and the number of controls per case | Methods / Study Population, Dementia Ascertainment | RECORD 6.1: The methods of study population selection (such as codes or algorithms used to identify subjects) should be listed in detail. If this is not possible, an explanation should be provided.  RECORD 6.2: Any validation studies of the codes or algorithms used to select the population should be referenced. If validation was conducted for this study and not published elsewhere, detailed methods and results should be provided.  RECORD 6.3: If the study involved linkage of databases, consider use of a flow diagram or other graphical display to demonstrate the data linkage process, including the number of individuals with linked data at each stage. | Methods / Study Population, Dementia Ascertainment  Methods / Study Population, Dementia Ascertainment  Methods / Study Population . Study Flow Chart |  |
| Variables | 7 | Clearly define all outcomes, exposures, predictors, potential confounders, and effect modifiers. Give diagnostic criteria, if applicable. | Method / Dementia Ascertainment, Outcomes, Covariates | RECORD 7.1: A complete list of codes and algorithms used to classify exposures, outcomes, confounders, and effect modifiers should be provided. If these cannot be reported, an explanation should be provided. | Method/ Dementia Ascertainment, Outcomes, Covariates, Supplement C, D, E |  |
| Data sources/ measurement | 8 | For each variable of interest, give sources of data and details of methods of assessment (measurement).  Describe comparability of assessment methods if there is more than one group | Supplement A, C, D Methods / Outcome measures / Covariates |  |  |  |
| Bias | 9 | Describe any efforts to address potential sources of bias | Methods / Statistical Analysis, Supplement F |  |  |  |
| Study size | 10 | Explain how the study size was arrived at | Method/Data sources and procedures |  |  |  |
| Quantitative variables | 11 | Explain how quantitative variables were handled in the analyses. If applicable, describe which groupings were chosen, and why | Method / Dementia Ascertainment / Statistical Analyses / Supplement C, D, Supplement E |  |  |  |
| Statistical methods | 12 | (a) Describe all statistical methods, including those used to control for confounding  (b) Describe any methods used to examine subgroups and interactions  (c) Explain how missing data were addressed  (d) *Case-control study* - If applicable, explain how matching of cases and controls was addressed  (e) Describe any sensitivity analyses | (a) Methods / Statistical Analysis, Supplement F  (b) Methods / Statistical Analysis  (c) Methods / Statistical Analysis  (d) Methods / Statistical Analysis Supplement F  (e) N/A | | |  |
| Data access and cleaning methods |  | .. |  | RECORD 12.1: Authors should describe the extent to which the investigators had access to the database population used to create the study population.  RECORD 12.2: Authors should provide information on the data cleaning methods used in the study. | Authors contribution  Methods / Database and procedures  Study flow chart |  |
| Linkage |  | .. |  | RECORD 12.3: State whether the study included person-level, institutional-level, or other data linkage across two or more databases. The methods of linkage and methods of linkage quality evaluation should be provided. | Methods / Databases |  |
| **Results** | | | | | | |
| Participants | 13 | (a) Report the numbers of individuals at each stage of the study (*e.g.*, numbers potentially eligible, examined for eligibility, confirmed eligible, included in the study, completing follow-up, and analysed)  (b) Give reasons for non-participation at each stage.  (c) Consider use of a flow diagram | Results/Demographic and sample characteristic  Study flow chart | RECORD 13.1: Describe in detail the selection of the persons included in the study (*i.e.,* study population selection) including filtering based on data quality, data availability and linkage. The selection of included persons can be described in the text and/or by means of the study flow diagram. | Results/Demographic and sample characteristic  Study flow chart |  |
| Descriptive data | 14 | (a) Give characteristics of study participants (*e.g.*, demographic, clinical, social) and information on exposures and potential confounders  (b) Indicate the number of participants with missing data for each variable of interest | (a, b) Table 1  Supplement F |  |  |  |
| Outcome data | 15 | *Case-control study* - Report numbers in each exposure category, or summary measures of exposure  *Cross-sectional study* - Report numbers of outcome events or summary measures | Table 1 |  |  |  |
| Main results | 16 | (a) Give unadjusted estimates and, if applicable, confounder-adjusted estimates and their precision (e.g., 95% confidence interval). Make clear which confounders were adjusted for and why they were included  (b) Report category boundaries when continuous variables were categorized  (c) If relevant, consider translating estimates of relative risk into absolute risk for a meaningful time period | Table 2, Table 3  Table 1  Not applicable |  |  |  |
| Other analyses | 17 | Report other analyses done—e.g., analyses of subgroups and interactions, and sensitivity analyses | Results, Supplement G, Supplement I |  |  |  |
| **Discussion** | | | | | | |
| Key results | 18 | Summarise key results with reference to study objectives | Discussion 1^st^ Paragraph |  |  |  |
| Limitations | 19 | Discuss limitations of the study, taking into account sources of potential bias or imprecision. Discuss both direction and magnitude of any potential bias | Discussion / Limitations | RECORD 19.1: Discuss the implications of using data that were not created or collected to answer the specific research question(s). Include discussion of misclassification bias, unmeasured confounding, missing data, and changing eligibility over time, as they pertain to the study being reported. | Discussion / Limitations |  |
| Interpretation | 20 | Give a cautious overall interpretation of results considering objectives, limitations, multiplicity of analyses, results from similar studies, and other relevant evidence | Discussion Limitations |  |  |  |
| Generalisability | 21 | Discuss the generalisability (external validity) of the study results | Discussion, 1^st^ section |  |  |  |
| **Other Information** | | | | | | |
| Funding | 22 | Give the source of funding and the role of the funders for the present study and, if applicable, for the original study on which the present article is based | Funding |  |  |  |
| Accessibility of protocol, raw data, and programming code |  | .. |  | RECORD 22.1: Authors should provide information on how to access any supplemental information such as the study protocol, raw data, or programming code. | N/A |  |

*Reference: Benchimol EI, Smeeth L, Guttmann A, Harron K, Moher D, Petersen I, Sørensen HT, von Elm E, Langan SM, the RECORD Working Committee. The REporting of studies Conducted using Observational Routinely-collected health Data (RECORD) Statement. *PLoS Medicine* 2015; in press.

*Checklist is protected under Creative Commons Attribution ([CC BY](http://creativecommons.org/licenses/by/4.0/)) license.

**References**

**Buckman, J E J, Saunders, R, Stott, J, Arundell, L L, O'driscoll, C, Davies, M R, Eley, T C, Hollon, S D, Kendrick, T, Ambler, G, Cohen, Z D, Watkins, E, Gilbody, S, Wiles, N, Kessler, D, Richards, D, Brabyn, S, Littlewood, E, Derubeis, R J, Lewis, G & Pilling, S** (2021a) Role of age, gender and marital status in prognosis for adults with depression: An individual patient data meta-analysis. *Epidemiol Psychiatr Sci,* 30**,** e42.

**Buckman, J E J, Saunders, R, Stott, J, Cohen, Z D, Arundell, L-L, Eley, T C, Hollon, S D, Kendrick, T, Ambler, G, Watkins, E, Gilbody, S, Kessler, D, Wiles, N, Richards, D, Brabyn, S, Littlewood, E, Derubeis, R J, Lewis, G & Pilling, S** (2022) Socioeconomic Indicators of Treatment Prognosis for Adults With Depression: A Systematic Review and Individual Patient Data Meta-analysis. *JAMA Psychiatry,* 79**,** 406-416.

**Buckman, J E J, Stott, J, Main, N, Antonie, D M, Singh, S, Naqvi, S A, Aguirre, E, Wheatley, J, Cirkovic, M, Leibowitz, J, Cape, J, Pilling, S & Saunders, R** (2021b) Understanding the psychological therapy treatment outcomes for young adults who are not in education, employment, or training (NEET), moderators of outcomes, and what might be done to improve them. *Psychological Medicine***,** 1-12.

**Huusko, J, Tuominen, S, Studer, R, Corda, S, Proudfoot, C, Lassenius, M & Ukkonen, H** (2020) Recurrent hospitalizations are associated with increased mortality across the ejection fraction range in heart failure. *ESC Heart Failure,* 7**,** 2406-2417.

**Langworthy, B, Wu, Y & Wang, M** (2022) An overview of propensity score matching methods for clustered data. *Statistical Methods in Medical Research,* 32**,** 641-655.

**The Improving Access to Psychological Therapies Manual** National Collaborating Centre for Mental Health**.** 2021a. [Online]. Available: <https://www.england.nhs.uk/wp-content/uploads/2018/06/the-iapt-manual-v5.pdf> [Accessed 2022].

**National Collaborating Centre for Mental Health** (2021b) The Improving Access to Psychological Therapies Manual. National Collaborating Centre for Mental Health.

**National Collaborating Centre for Mental Health** (2025) NHS talking therapies for anxiety and depression manual.

**Linked HES-ONS mortality data** Nhs Digital**.** 2020. [Online]. Available: <https://digital.nhs.uk/data-and-information/data-tools-and-services/data-services/linked-hes-ons-mortality-data> [Accessed].

**Hospital Episode Statistics (HES)** Nhs Digital**.** 2021a. [Online]. Available: <https://digital.nhs.uk/data-and-information/data-tools-and-services/data-services/hospital-episode-statistics> [Accessed].

**Mental Health Services Data Set** Nhs Digital**.** 2021b. [Online]. Available: <https://digital.nhs.uk/data-and-information/data-collections-and-data-sets/data-sets/mental-health-services-data-set> [Accessed].

**Saunders, R, Buckman, J E J, Stott, J, Leibowitz, J, Aguirre, E, John, A, Lewis, G, Cape, J & Pilling, S** (2021) Older adults respond better to psychological therapy than working-age adults: evidence from a large sample of mental health service attendees. *Journal of Affective Disorders,* 294**,** 85-93.

**Saunders, R, Cape, J, Leibowitz, J, Aguirre, E, Jena, R, Cirkovic, M, Wheatley, J, Main, N, Pilling, S & Buckman, J E J** (2020) Improvement in IAPT outcomes over time: are they driven by changes in clinical practice? *Cogn Behav Therap,* 13**,** e16.
